# Golgi enzymes are retrieved from the plasma membrane to the trans‐Golgi network

**DOI:** 10.1002/1873-3468.70438

**Published:** 2026-08-15

**Authors:** Dominik P. Buser, Tina Junne

**Affiliations:** ^1^ Biozentrum University of Basel Switzerland

**Keywords:** endosome, Golgi enzymes, Golgi‐IP, microscopy, nanobodies, retrograde transport, sulfation, trans‐Golgi network

## Abstract

The Golgi complex is not only a central hub for bidirectional protein trafficking, but also a key site for post‐translational modifications; the enzymes responsible are trafficked to the Golgi. To determine whether Golgi‐resident enzymes can be recaptured from distal sites, we employed a nanobody‐based transport assay. While most enzymes studied localized predominantly to the Golgi complex, some were also observed at the plasma membrane. Using sulfation‐competent and fluorescently marked nanobody, we observed that certain enzymes are capable of retrograde transport to the trans‐Golgi network. In contrast, classical and abundant recycling receptors readily internalized the nanobody but did not undergo retrograde transport. Our findings provide evidence that select Golgi enzymes can be retrieved from the cell surface to the sulfation compartment.

## Graphical abstract

Golgi enzymes are traditionally considered resident proteins retained within the Golgi apparatus. Here, we demonstrate that a subset transiently reaches the cell surface and is subsequently retrieved to the trans‐Golgi network via retrograde transport. Using a nanobody‐based toolkit, we uncover a dynamic trafficking cycle of several Golgi enzymes.

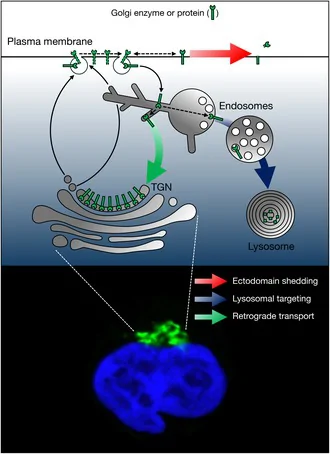

## Abbreviations

**B3GalT6**, β‐1,3‐galactosyltransferase 6

**BAP**, biotin acceptor peptide

**CD59**, CD59 glycoprotein (protectin; GPI‐anchored complement regulatory protein)

**CD8**, cluster of differentiation 8

**CDMPR**, cation‐dependent mannose‐6‐phosphate receptor

**CNX**, calnexin

**CTSD**, cathepsin D

**CTSZ**, cathepsin Z

**EEA1**, early endosome antigen 1

**ERGIC53**, ER‐Golgi intermediate compartment protein 53 (also LMAN1)

**FR1**, folate receptor 1

**FucT**, fucosyltransferase 6

**GALNT2**, polypeptide N‐acetylgalactosaminyltransferase 2

**GalT**, β‐1,4‐galactosyltransferase

**GM130**, Golgi matrix protein of 130 kDa

**GOLM1**, Golgi membrane protein 1 (also known as GP73 or GOLPH2)

**H1**, asialoglycoprotein receptor subunit H1

**HA**, hemagglutinin epitope tag

**LAMP1**, lysosome‐associated membrane protein 1

**Man1B1**, ER α‐1,2‐mannosidase

**ManII**, α‐mannosidase II

**MGAT1**, N‐acetylglucosaminyltransferase I

**MGAT2**, N‐acetylglucosaminyltransferase II

**POMGNT1**, protein O‐linked mannose N‐acetylglucosaminyltransferase 1

**RCAS1**, Receptor‐binding Cancer Antigen expressed on SiSo cells 1

**SiaT**, α‐2,6‐sialyltransferase

**SPEx**, Subcellular spatial Proteomics coupled to Expansion

**SPG11**, spastic paraplegia 11/Spatacsin

**SPG15**, spastic paraplegia 15/Spastizin

**tev**, tobacco etch virus protease cleavage site

**TfR**, transferrin receptor

**TGN46**, trans‐Golgi network protein 46

**TMEM115**, transmembrane protein 115

**TMEM192**, transmembrane protein 192

**TPST1**, tyrosylprotein sulfotransferase 1

**TPST2**, tyrosylprotein sulfotransferase 2

**VHH**, variable domain of heavy‐chain‐only antibody

The Golgi complex is the major processing and sorting station at the crossroads of the biosynthetic pathway. It receives newly synthesized soluble secretory and transmembrane proteins from the endoplasmic reticulum (ER), modifies them using Golgi‐localizing enzymes, and sorts them via the trans‐Golgi network (TGN) to the endolysosomal system or the plasma membrane (PM). Proper localization of the modification machinery to the Golgi complex is not only critical for the homeostasis of this organelle but also for maturing and processing selected cargo that travels through.

The majority of Golgi‐resident enzymes are type II transmembrane proteins characterized by a modular domain architecture consisting of a relatively short N‐terminal cytosolic tail (NCT), a single transmembrane domain (TMD), and an extracellular domain (ED). The ED, in turn, is composed of two structurally and functionally distinct regions: a juxtamembrane stem segment and a more distal catalytic domain. Cumulative mechanistic and mutagenesis‐based studies suggest that each of these domains—including the N‐terminal tail—contributes critically to the proper sub‐Golgi localization and retention of these enzymes [1]. To explain the principles underlying their selective localization and restricted distribution across the secretory pathway, three prevailing models have been proposed: the kin‐recognition model, the TMD length‐based sorting model, and the cytosolic sorting motif model [1].

The kin‐recognition model posits that Golgi enzymes form large homo‐ and/or hetero‐oligomeric complexes or aggregates within the Golgi, thereby preventing their onward transport to post‐Golgi compartments [2]. Supporting this view, numerous Golgi enzymes have been found to exist in high molecular weight complexes in membrane fractions isolated from cells [3]. However, findings that forced oligomerization of mannosidase I or galactosyltransferase via FKBP12 domains result in lysosomal targeting argue against this model as a general retention mechanism [4].

According to the TMD‐based sorting model, Golgi residency is determined by the hydrophobic length of the transmembrane domain [5]. This hypothesis is grounded in the observation that single‐pass transmembrane proteins tend to partition into lipid bilayer regions whose thickness optimally accommodates their TMDs. Membrane thickness increases progressively along the secretory pathway and this gradient correlates with the average TMD lengths of proteins residing in the respective compartments [6]. In support of this model, many Golgi enzymes harbor shorter TMDs than their counterparts localized to the plasma membrane, such as the transferrin receptor (TfR) or the asialoglycoprotein receptor (ASGPR) subunit H1. However, notable exceptions such as TGN46—a type I transmembrane protein that efficiently localizes to the Golgi regardless of whether its TMD is shortened, wild‐type, or replaced with a heterologous sequence [7]—suggest that TMD length alone is not the sole determinant of Golgi localization.

Finally, the cytosolic sorting motif model proposes that the cisternal distribution of Golgi enzymes is governed by specific sequence motifs within their cytosolic tails. These motifs serve as recognition elements for vesicle coat components that mediate active sorting. For example, the NCTs of several Golgi enzymes have been shown to directly interact with the δ‐ and ζ‐subunits of the COPI coatomer complex, whereas others rely on the adaptor protein GOLPH3 to facilitate indirect interaction with COPI [8, 9, 10].

The three prevailing models further raise the question of whether retention or retrieval constitutes the dominant mechanism governing Golgi localization. Retention refers to the ability of proteins to remain confined within their appropriate organelle or subcompartment, mediated by specific sequence motifs, membrane characteristics, or molecular interactions that preclude their inadvertent export via carriers. Conversely, retrieval involves the active return of proteins that have escaped their native compartment, utilizing signal‐dependent retrograde transport carriers to restore organelle identity and prevent protein mislocalization or unintended leakage. Whether such signal‐dependent sorting mechanisms—exemplified by COPI‐mediated intra‐Golgi transport—also apply to the retrieval of trans‐Golgi/TGN‐resident enzymes from post‐Golgi membranes remains an open and addressable question.

Although Golgi enzymes predominantly localize to the Golgi complex, a minor fraction resides within the endoplasmic reticulum. Moreover, small but detectable amounts of both endogenous and overexpressed Golgi enzymes have been observed at the plasma membrane [11], raising the possibility that post‐Golgi trafficking of these proteins may be biologically relevant. Intriguingly, a recent study reported a selection of Golgi enzymes to locate to the cell surface [12]. Most of the assayed enzymes, however, were not substantially retrieved to the Golgi complex, but rather targeted to lysosomes. Their observation suggested a model in which retention, rather than machinery‐dependent retrieval, governs Golgi enzyme localization [12]. This interpretation, however, can be challenged by findings that several canonical ‘Golgi residents’ with type II membrane topology are among the most significantly depleted proteins in vesicle‐enriched fractions of adaptor protein (AP)‐5 complex knockout cells [13]. AP‐5 is one of several retrograde trafficking modules that mediate endosome‐to‐Golgi transport [14]. Further, clathrin‐coated vesicles (CCVs) isolated from HeLa cells with rapidly inactivating AP‐1 and epsinR by knocksideways also showed a depletion of some Golgi residents in the retrograde or anterograde pathway [15].

Given that even a minor contribution of retrieval cannot be dismissed as a determinant for Golgi residency, we set out to test the potential of various Golgi enzymes to undergo retrograde transport from the PM to the TGN. To this end, we established stable cell lines expressing a panel of EGFP‐tagged reporter proteins, including the well‐characterized retrograde cargo receptor cation‐dependent mannose‐6‐phosphate receptor (CDMPR), the recycling endocytic receptor transferrin receptor (TfR), as well as several Golgi‐resident enzymes such as β‐1,4‐galactosyltransferase I (GalT), α‐2,6‐sialyltransferase I (SiaT), α‐mannosidase II (ManII), and tyrosylprotein sulfotransferases 1 and 2 (TPST1 and TPST2). To quantitatively assess PM‐to‐TGN transport of these fusion proteins, we employed our previously established nanobody‐based Golgi arrival assay, which relies on anti‐GFP nanobodies engineered with tyrosine sulfation (TS) sequences [16]. Sulfation of nanobody cargo serves as a biochemical proxy for arrival at the TGN, the site of sulfotransferase activity. As expected, nanobodies trafficked and carried by CDMPR were efficiently sulfated, while those associated with TfR remained unsulfated. Strikingly, when functionalized nanobodies were applied to cells expressing GFP‐tagged Golgi enzymes, a substantial degree of nanobody sulfation was observed, suggesting selective retrograde transport of these enzymes to the TGN. To further substantiate these findings, we employed fluorescently labeled anti‐GFP nanobodies to validate TGN arrival of Golgi enzyme–nanobody complexes by fluorescence microscopy. We reinforced our observations by extending the analysis to an additional panel of Golgi reporter constructs. Last but not least as a third and independent approach to validate Golgi arrival of surface‐accessible Golgi enzymes, we employed Golgi‐IP, a method that enables the selective enrichment of highly purified Golgi membranes.

Based on these findings, we propose the existence of an active, cargo‐specific recycling pathway that facilitates the retrieval of Golgi enzymes from the cell surface, thereby contributing to the maintenance of Golgi identity and function.

## Results and Discussion

### Recombinant Golgi enzymes have a predominant Golgi localization

To investigate retrograde trafficking of proteins to intracellular compartments, including the TGN, we generated stable HeLa cell lines expressing a panel of EGFP‐tagged reporter proteins with either well‐characterized or undefined intracellular itineraries. These included the transferrin receptor (TfR), which cycles between the plasma membrane and early (sorting and recycling) endosomes, as well as a set of type II transmembrane Golgi‐resident enzymes: β‐1,4‐galactosyltransferase I (GalT), α‐2,6‐sialyltransferase I (SiaT), α‐mannosidase II (ManII), and tyrosylprotein sulfotransferases 1 and 2 (TPST1 and TPST2). As previously described and done for the TfR reporter construct (Buser et al., [16]; Buser and Spiess [17]), the EGFP moiety was fused to the carboxyterminal end of each Golgi reporter protein. This design ensured that the cytoplasmic tail—often containing critical sorting motifs—remained unaltered and accessible for potential interactions with sorting machinery, while the lumenal domain was oriented to permit nanobody recognition (Fig. 1A). Notably, TPST1‐EGFP and TPST2‐EGFP constructs comprised the full‐length proteins, including their enzymatic domains. In contrast, GalT‐EGFP, SiaT‐EGFP, and ManII‐EGFP reporters were truncated variants encompassing only their N‐terminal cytoplasmic tail (NCT), transmembrane domain (TMD), and juxtamembrane stem region—domains previously validated as suitable for studying Golgi membrane dynamics (Fig. 1A) [18].

**Fig. 1:**
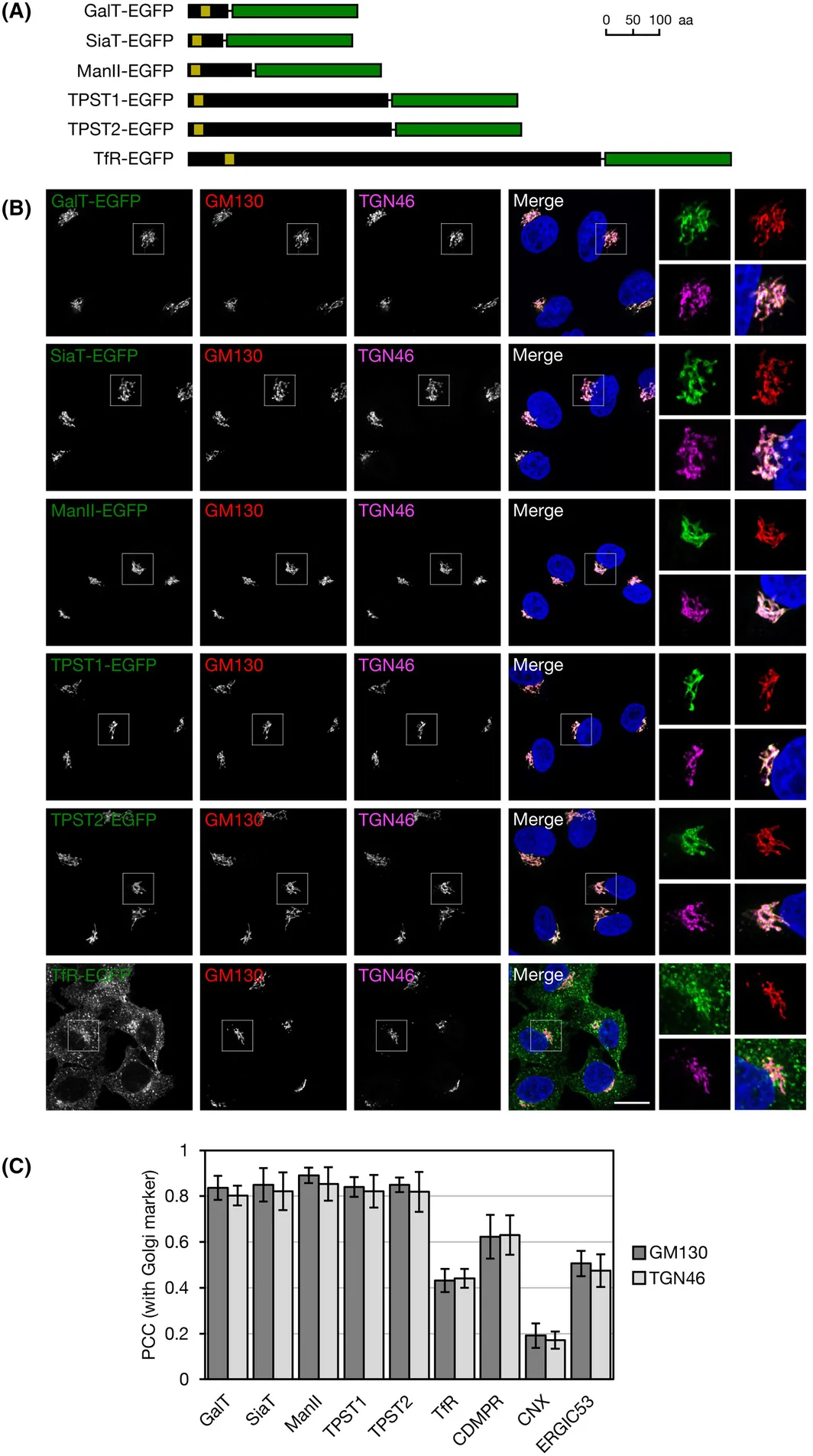
Localization of EGFP‐tagged Golgi enzymes to the Golgi compartment. (A) Schematic representation of EGFP (green) fusion proteins. Sequences derived from Golgi enzyme or receptor proteins are shown in black with internal transmembrane segments in yellow. EGFP was fused C‐terminally to full‐length TPST1, TPST2, and TfR, and to the cytoplasmic sequence and transmembrane segment of GalT, SiaT, and ManII. Scale bar in amino acids (aa). (B) HeLa cells stably expressing GalT‐, SiaT‐, ManII‐, TPST1‐, TPST2‐, and TfR‐EGFP were fixed, stained for GM130, TGN46, and nuclei (DAPI, blue), and imaged by fluorescence microscopy. Insets display enlarged regions of the selected area. For figure preparation, brightness was adjusted for visualization only. When applicable, all quantitative analyses were performed using the original, unprocessed image data. Bar: 20 μm. (C) Quantitative colocalization analysis of EGFP‐tagged Golgi enzymes or TfR, CDMPR, CNX, ERGIC53 with GM130 and TGN46. Pearson's correlation coefficients (PCC) were determined to quantify the extent of colocalization between EGFP‐tagged reporter or Golgi enzymes and GM130 or TGN46. For each condition, 50–60 cells from randomly selected frames were evaluated across three independent experiments. Data represent mean values ± standard deviation from three biological replicates.

To validate the predominant Golgi localization of our EGFP‐tagged Golgi reporters, we examined their steady‐state distribution by fixed‐cell fluorescence microscopy and their expression by western blotting (Fig. 1B and Fig. S1C,D). Golgi compartmentalization was assessed through colocalization analysis with the endogenous Golgi marker proteins GM130 and TGN46. As anticipated based on their established function, recombinantly expressed Golgi enzymes exhibited a pronounced overlap with these reference markers, yielding Pearson correlation coefficients (PCC) greater than 0.8 (Fig. 1C). In contrast, the transferrin receptor—which transits through the Golgi along the biosynthetic pathway en route to the endosomal system and the plasma membrane—showed only minimal spatial overlap with the antibody‐labeled Golgi markers (Fig. 1B,C). As an additional reference, we included our workhorse reporter EGFP‐CDMPR. Since MPRs are involved in the efficient transport of newly synthesized lysosomal hydrolases from the TGN to endosomes, it is not surprising to detect a notable, albeit less pronounced, colocalization with GM130 and TGN46. This observation is consistent with previous reports indicating a dual steady‐state distribution of EGFP‐CDMPR between the TGN and endosomal compartments [19] (Fig. 1C and Fig. S1A,B). To expand our panel of controls for retrograde trafficking assessments, we also included EGFP‐tagged Calnexin (CNX) and ERGIC53. As expected, CNX exhibited no extensive overlap, and ERGIC53 only partial colocalization with Golgi markers, as quantified by PCC analysis (Fig. 1C and Fig. S1A,B). Collectively, our immunofluorescence analyses confirm that all EGFP‐tagged Golgi enzyme reporters exhibit robust and highly specific localization to the Golgi apparatus.

### Ultrastructural localization of recombinant Golgi enzymes

To further substantiate the predominant localization of recombinant Golgi enzymes within the Golgi subcompartments, we subjected the same panel of reporter constructs (Fig. 1A) to immunoelectron microscopy. GalT, SiaT, TPST1, and TPST2 have been previously reported to localize to the trans face of the Golgi complex, encompassing both the trans‐Golgi cisternae and the trans‐Golgi network in mammalian cells [20]. Conversely, ManII is well‐established to reside predominantly in the medial cisternae of the Golgi stack [21]. Consistent with our fluorescence microscopy findings, immunoelectron microscopy revealed a pronounced Golgi enrichment for all enzymes examined (Fig. 2A). Utilizing an indirect immunogold labeling approach, we observed that ~ 85% of the gold particles associated with the Golgi complex (Fig. 2B). This distribution mirrors earlier findings indicating a steady‐state partitioning of glycosyltransferases between the Golgi and ER in a ratio of approximately 90 : 10 [22]. The ER, beyond serving as the site of enzyme biosynthesis and folding, may also act as a transient recycling station for Golgi enzymes after retrograde Golgi‐to‐ER transport, in particular during mitosis [18]. Nevertheless, this view has been challenged by reports arguing against substantial Golgi‐to‐ER mixing of Golgi enzymes under both interphase and mitotic conditions [23]. Occasionally, gold particles—particularly for enzymes with known trans‐Golgi/TGN localization—were also detected at peripheral locations, hinting at post‐Golgi trafficking (Fig. 2A). Notably, GalT has been reported not only to localize to the Golgi and ER, but also to the plasma membrane (PM), where it can function as a matrix receptor [24]. Despite this, our electron microscopy data robustly support the predominant localization of these enzymes within the Golgi apparatus.

**Fig. 2:**
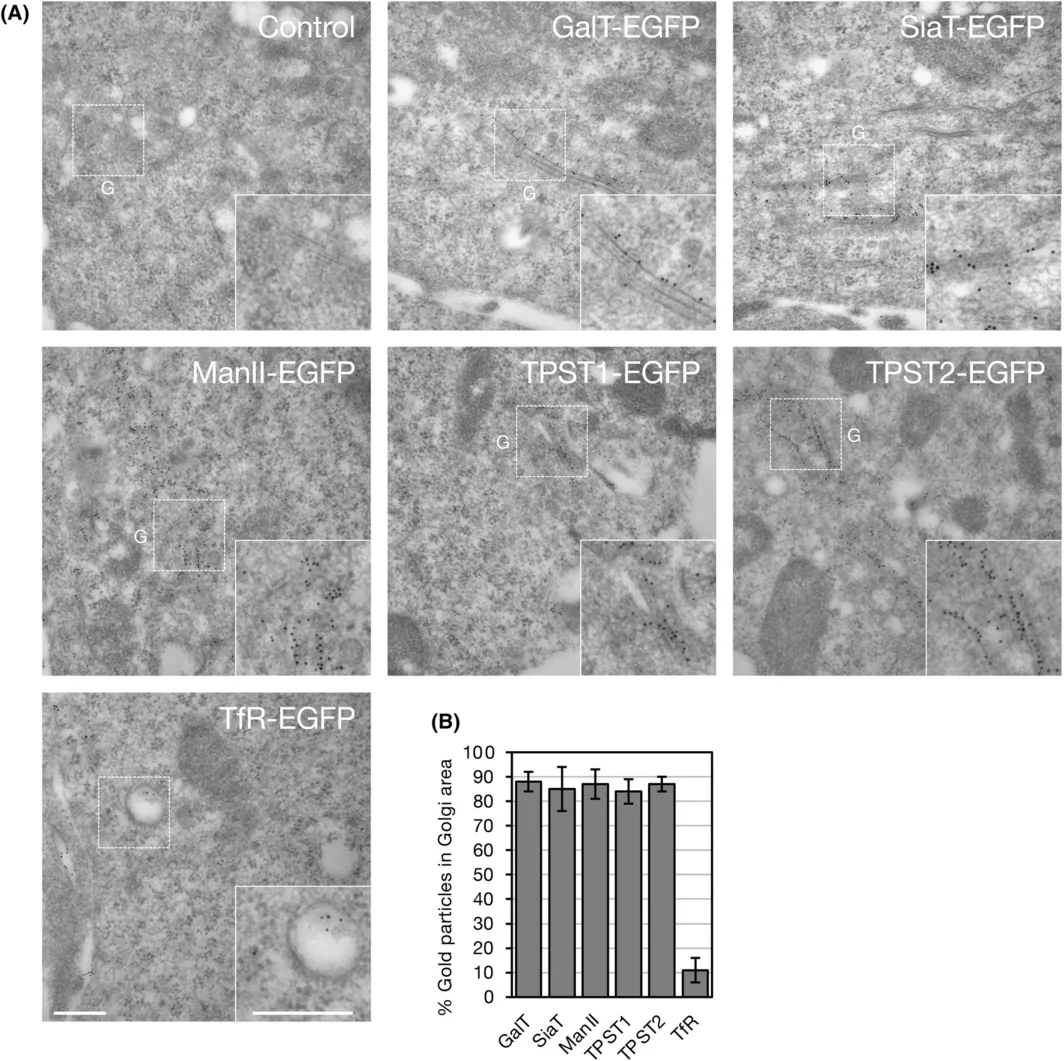
Immunogold electron microscopy of EGFP‐tagged Golgi enzymes shows their predominant Golgi localization. (A) Immunoelectron microscopy of HeLa cells stably expressing GalT‐, SiaT‐, ManII‐, TPST1‐, TPST2‐, and TfR‐EGFP. HeLa expressing no reporter protein was processed as control. Sections were labeled with anti‐GFP antibodies, followed by secondary labeling with an antibody coupled to 10‐nm colloidal gold. Insets display enlarged regions of the Golgi area. G indicates Golgi membranes/area. Bar: 500 nm. (B) Quantitative localization analysis of gold particles in the Golgi area. For each condition, 10–13 random sections from cells were evaluated based on gold particle proximity to the Golgi. Data represent mean values ± standard deviation from three biological replicates.

Recently, several tagged glycosyltransferases, including SiaT and TPST isoforms, were probed for surface exposure using either our engineered anti‐GFP nanobodies or antibodies recognizing common epitope tags [12]. Most of these enzymes were found to be accessible to extracellular binders, confirming their post‐Golgi export and subsequent plasma membrane arrival [12]. That study also revealed that the majority of escaped enzymes are subject to extracellular domain shedding or lysosomal targeting, supporting a model in which retention, rather than retrieval, serves as the principal mechanism ensuring Golgi localization [12]. However, the physiological rationale for Golgi enzyme appearance at the cell surface remains unclear. Is their ectopic presence at the plasma membrane functionally analogous to GalT, or does it rather mark the terminal phase of their lifespan, designating them for degradation? Alternatively, might the Golgi complex, akin to the ER, display a degree of stochastic leakiness, whereby protein egress is subsequently counteracted by retrieval to restore homeostasis? In the ER, lumenal residents bear the KDEL motif, which facilitates their recognition by H/KDEL receptors and subsequent retrieval from the Golgi, thereby safeguarding organelle identity and suppressing the unfolded protein response [25]. By analogy, Golgi enzymes escaping their native compartment may disrupt cisternal enzymatic processing and compromise organellar function. This led us to question whether such Golgi enzymes can, in fact, be recycled from the cell surface to the TGN.

### Sulfatable nanobodies reveal cell surface‐to‐TGN transport of Golgi enzymes

To evaluate whether Golgi‐resident enzymes can be retrieved from the cell surface to the TGN, we employed our previously established, versatile toolkit of functionalized anti‐GFP nanobodies [26]. This approach enables the labeling of EGFP‐tagged surface proteins and tracking their intracellular routes through endosomes, the TGN, and back to the plasma membrane. For the specific detection of TGN arrival, we harnessed tyrosine sulfation—a post‐translational modification exclusively occurring in the trans‐Golgi/TGN [27]. To this end, we utilized anti‐GFP nanobodies (VHHs, the variable domain of camelid homodimeric heavy‐chain‐only antibodies) modified with a hexahistidine tag for purification, T7 and HA epitope tags for immunodetection, a biotin acceptor peptide for *in vivo* biotinylation, a TEV protease cleavage site, and four tandem tyrosine sulfation motifs (VHH‐tev‐4xTS) (Fig. 3A). These nanobodies were expressed in *E. coli*, purified to homogeneity, and validated for efficient epitope detection using western blotting (Fig. 3B,C).

**Fig. 3:**
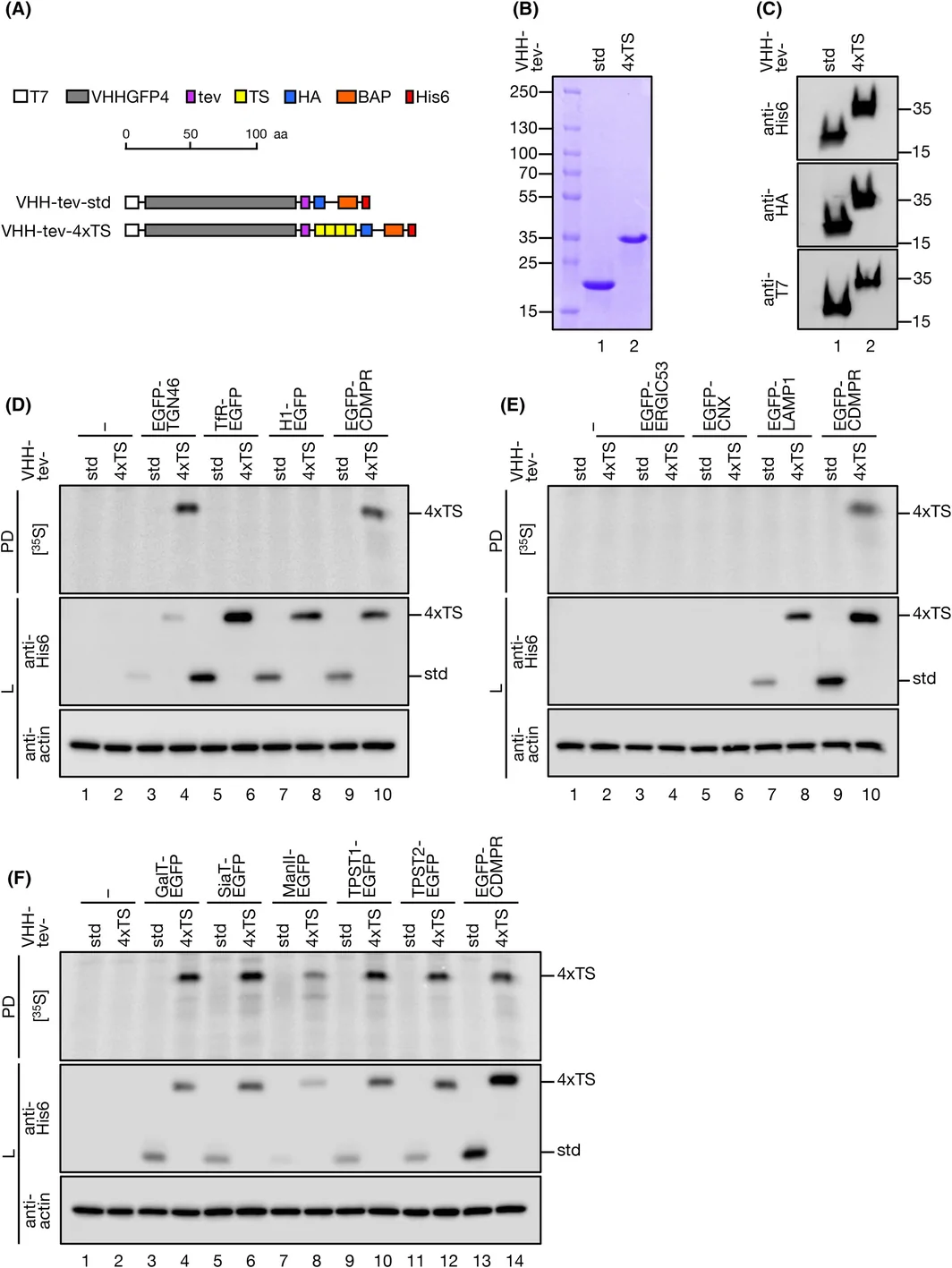
Retrograde transport analysis of Golgi enzymes and reporters using sulfatable nanobodies. (A) Schematic representation of the derivatized nanobodies. The modified standard nanobody (VHH‐tev‐std) consists of the GFP‐specific VHH domain, T7 and HA epitope tags, a TEV cleavage site, a BAP, and a hexahistidine (His6) purification tag. The other nanobody for studying retrograde transport in addition contains four consensus sequences for tyrosine sulfation. Scale bar is in amino acids (aa). (B) Bacterially expressed and purified nanobodies (30 μg) were analyzed by SDS‐gel electrophoresis and Coomassie stained. Marker proteins with molecular mass in kiloDaltons are shown on the left. (C) Immunoblot analysis of nanobodies (30 ng) with antibodies against the T7, HA, or His6 epitopes. Marker proteins with molecular mass in kiloDaltons are shown on the left. (D–F) HeLa cells stably expressing the indicated EGFP‐tagged membrane proteins were labeled with [^35^S]sulfate for 60 min with 2 μg·mL^−1^ VHH‐tev‐std or VHH‐tev‐4xTS. HeLa expressing no reporter protein was processed as control. The nanobodies were isolated using Nickel resin‐based pulldowns (PD) and subjected to autoradiography ([^35^S]). A fraction of the postnuclear supernatant of lysed cells after labeling was subjected to immunoblot analysis. Aliquots of collected postnuclear cell lysates (L) were immunoblotted for His6 (total cell‐associated nanobody) and actin (loading control).

To systematically assess TGN arrival, we extended our analysis to include EGFP‐tagged reporter proteins with well‐characterized trafficking itineraries. As a model of a classical recycling receptor, we not only included TfR, but also the asialoglycoprotein receptor subunit H1 [28], C‐terminally fused to EGFP (Fig. S2A,B). Additionally, we examined lysosome‐associated membrane protein 1 (LAMP1), a transmembrane protein known to transiently appear at the plasma membrane en route to lysosomes [29] (Fig. S2A,B). Importantly, neither H1 nor LAMP1 has been reported to undergo retrograde transport to the TGN. To rule out the possibility that reporter surface expression results from overexpression artifacts or transport machinery pathway saturation, we also included EGFP‐CNX and EGFP‐ERGIC53, previously shown not to appear at the plasma membrane [17], thus serving as robust negative controls for nanobody binding and import.

Stable HeLa cell lines expressing these EGFP reporters were incubated with VHH‐tev‐4xTS nanobodies for 60 min in the presence of radioactive [^35^S]sulfate. Subsequently, nanobody‐bound fractions were isolated using Nickel beads and analyzed by immunoblotting and autoradiography. While immunoblotting confirmed nanobody uptake, autoradiographic detection of incorporated [^35^S]sulfate reported TGN arrival. As previously observed, nanobodies imported by EGFP‐TGN46 and EGFP‐CDMPR were efficiently sulfated [16] (Fig. 3D). Interestingly, the sulfation‐to‐uptake ratio of nanobody was notably higher for TGN46 than for CDMPR, in part suggesting a longer nanobody residence time at the TGN for the former (Fig. 3D). In contrast, TfR‐EGFP and H1‐EGFP captured significantly more nanobody at the cell surface but failed to deliver it to the sulfation‐competent compartment, corroborating their noninvolvement in retrograde trafficking [16] (Fig. 3D). Similarly, EGFP‐LAMP1 did not support nanobody sulfation, despite its distinct sorting itinerary through the late secretory pathway (Fig. 3E). As expected, CNX and ERGIC53 did not bind or internalize nanobodies, confirming their absence from the plasma membrane under the conditions tested (Fig. 3E).

Strikingly, when VHH‐tev‐4xTS nanobodies were administered to cells expressing EGFP‐tagged Golgi enzymes, we observed robust and reporter‐specific nanobody sulfation already after 1 h (Fig. 3F). These data provide evidence that assayed Golgi‐resident enzymes undergo active retrograde transport from the plasma membrane back to the TGN. The specificity and consistency of sulfation argue against passive transport or stochastic mislocalization and instead point to a machinery‐driven retrieval mechanism. Relative to CDMPR, Golgi enzymes demonstrated a greater increase in sulfation signal per internalized nanobody, whereas cargo proteins that do not undergo retrograde transport exhibited sulfation‐to‐uptake ratios beyond a background (Fig. 3F). The consistently elevated sulfation‐to‐uptake ratios observed for the Golgi enzyme reporters thus provide robust evidence for their efficient and selective retrograde delivery to the TGN.

The observation that components of the tyrosine sulfation machinery, namely TPST1 and TPST2, exit the Golgi complex and undergo retrieval may initially seem paradoxical, particularly given that we employ nanobody sulfation as a quantitative readout for arrival at the target compartment. This raises the concern that tyrosine sulfation, as a post‐translational modification, might itself become redistributed or mislocalized. However, it is essential to consider that the enzymatic activity of TPSTs is dependent on additional factors, such as transporters responsible for importing 3′‐phosphoadenosine‐5′‐phosphosulfate (PAPS) from the cytosol into the Golgi lumen. Notably, one such transporter, SLC35B2, has been shown to naturally exit the trans‐Golgi network and to be recycled via AP‐1‐ and AP‐5‐dependent pathways [15]. This coordinated cycling may serve as a safeguard mechanism to ensure functional integrity of sulfation within the TGN, even when key components transiently leave the compartment. No appreciable alterations in the steady‐state localization of TPST1 or TPST2 were observed upon AP‐1 depletion, whether by knockdown or genetic knockout [26]. Additionally, subcompartmental environmental parameters, such as pH or ionic strength, are likely to play critical roles in maintaining the specificity and efficiency of tyrosine sulfation, thereby preventing ectopic enzymatic activity elsewhere in the cell.

### Fluorescent nanobodies reveal Golgi enzyme retrieval by quantitative microscopy

To further substantiate our findings of Golgi enzyme retrieval to the sulfation‐competent compartment, we employed our previously established anti‐GFP nanobody fused to mCherry (Fig. S3A–C) [17]. To determine whether internalized nanobody–cargo complexes are also directed toward the endolysosomal pathway, we monitored their localization not only at the Golgi (GM130) but also within early endosomes (EEA1) and late endosomes/lysosomes (LAMP1), using well‐characterized compartment‐specific markers. As references for nanobody distribution following endocytosis, we included EGFP‐CDMPR and TfR‐EGFP, whose trafficking itineraries are well defined. To quantitatively assess compartmental distribution of the nanobody signal, we applied a thresholded Manders' colocalization coefficient, measuring the fraction of the respective compartment marker signal that overlaps with the mCherry (nanobody) signal.

Following incubation of these cells with VHH‐tev‐mCherry for 1 h at 37 °C, nanobody internalized via the CDMPR reporter was predominantly localized to early endosomes and the Golgi, in accordance with previously characterized trafficking itineraries of MPRs (Fig. 4A,D,E) [16]. In contrast, fluorescent nanobody delivered through TfR‐mediated endocytosis was primarily confined to EEA1‐positive early endosomes and was largely excluded from GM130‐labeled Golgi compartments. These observations are consistent with our prior findings demonstrating the absence of cell surface‐to‐TGN transport by TfR (Fig. 4B,D,E) [17]. Notably, both receptors exhibited minimal colocalization with LAMP1‐positive compartments, as reflected by low Manders' coefficients, indicative of limited lysosomal targeting (Fig. 4A,B,F). Our quantitative colocalization analyses of nanobody distribution were further substantiated by the observation that cells stably expressing EGFP‐CD8‐Furin similarly displayed predominant nanobody accumulation within GM130‐positive compartments (Fig. 4D–F and Figs S2C, S3E) [30]. As an additional control for a surface protein not undergoing TGN‐directed retrieval upon endocytosis, we included the glycosylphosphatidylinositol (GPI)‐anchored reporter EGFP‐CD59 (cluster of differentiation 59). In this context, nanobody internalized via CD59 was primarily localized to EEA1‐positive endosomal structures, while remaining largely excluded from both Golgi and lysosomal compartments (Fig. 4D–F, Fig. S3D) [31, 32, 33].

**Fig. 4:**
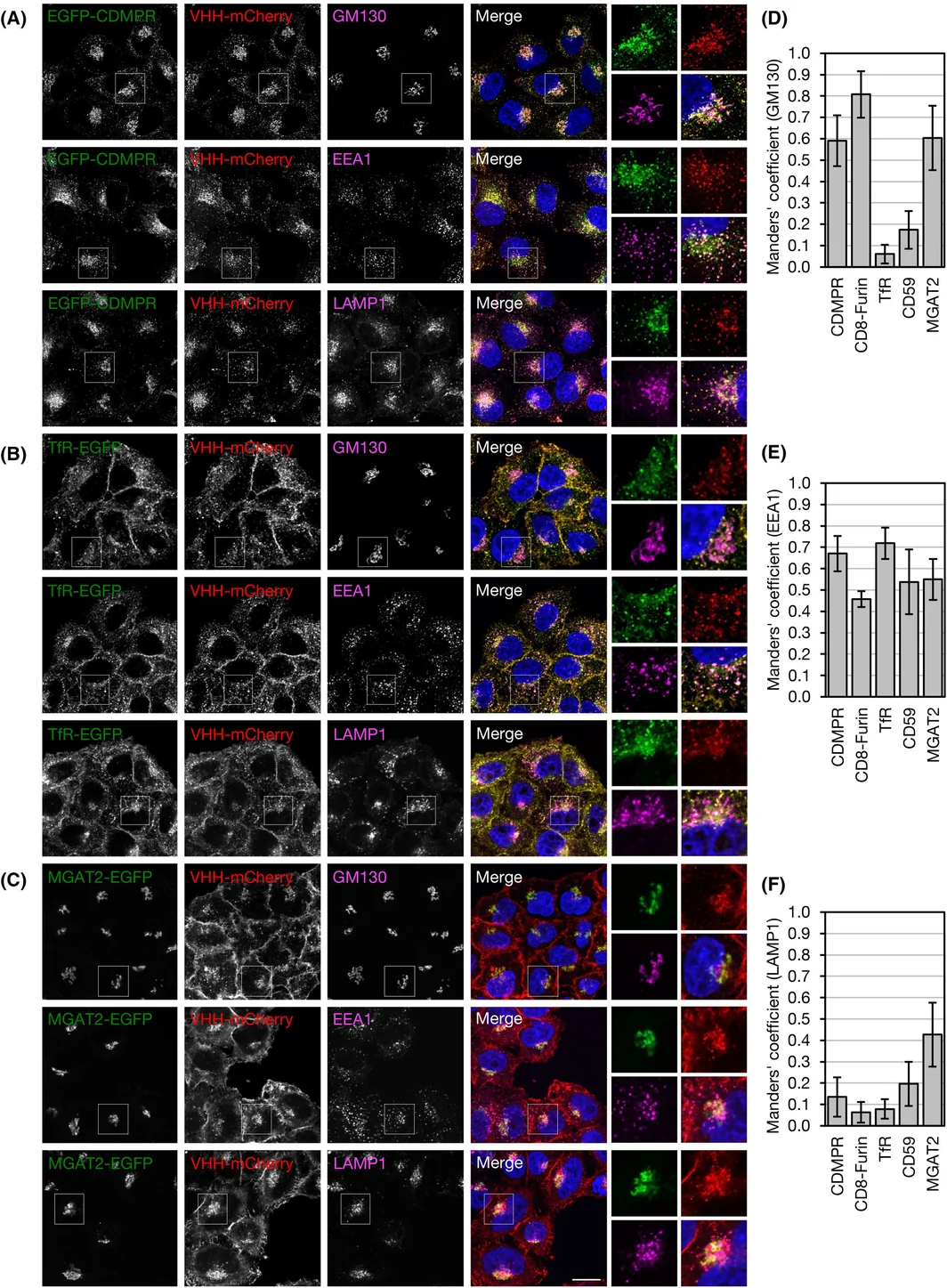
Quantitative compartment colocalization analysis of EGFP reporter‐mediated uptake of fluorescent nanobody. (A–C) HeLa cells stably expressing the indicated EGFP‐tagged membrane proteins were allowed to endocytose VHH‐tev‐mCherry (at 5 μg·mL^−1^) for 1 h (CDMPR and TfR) or 5 h (MGAT2), fixed, stained for GM130, EEA1, or LAMP1, and nuclei (DAPI, blue), and imaged by fluorescence microscopy. Insets display enlarged regions of the selected area. For figure preparation, brightness was adjusted for visualization only. When applicable, all quantitative analyses were performed using the original, unprocessed image data. Bar: 20 μm. (D–F) Quantitative compartment colocalization analysis of EGFP‐tagged CDMPR, CD8‐Furin, TfR, CD59, and MGAT2 with GM130 (Golgi), EEA1 (early endosomes), or LAMP1 (lysosomes). Manders' correlation coefficients (‘compartment signal’ in ‘mCherry signal’) were determined to quantify the extent of colocalization between EGFP‐tagged reporters and GM130, EEA1, or LAMP1. For each condition, 50–60 cells from randomly selected Z‐stack acquisitions were evaluated across three independent experiments. Data represent mean values ± standard deviation from three biological replicates.

Having established a nanobody‐based colocalization assay using robust cargo reporter proteins, we next sought to determine whether our sulfation‐based evidence for Golgi enzyme retrieval to the TGN could be independently validated. One Golgi enzyme previously shown to undergo retrieval to the TGN using a comparable microscopy‐based approach is MGAT2 [12]. We therefore incubated cells stably expressing MGAT2‐EGFP with VHH‐tev‐mCherry for 5 h at 37 °C to allow efficient internalization. We observed a comparable extent of nanobody accumulation within GM130‐positive compartments as detected for EGFP‐CDMPR, thereby confirming MGAT2 retrieval to the Golgi complex (Fig. 4C,D). Notably, trafficking of MGAT2‐EGFP to the Golgi did not appear to represent the exclusive postendocytic route. A substantial fraction of nanobody internalized via recombinant MGAT2 localized to LAMP1‐positive structures, suggesting that a portion of the internalized Golgi enzyme is directed toward lysosomal compartments (Fig. 4C,F). Consistent with the microscopy‐based evidence of MGAT2 retrieval to the Golgi (Fig. 4C), we detected robust sulfation of VHH‐tev‐4xTS in cells expressing MGAT2‐EGFP, whereas no detectable nanobody sulfation was observed in cells expressing GPI‐anchored reporter proteins (EGFP‐CD59 or EGFP‐FR1; folate receptor 1) (Fig. S3F,G).

We next investigated whether our GFP‐tagged Golgi enzymes—GalT, SiaT, ManII, TPST1, and TPST2—exhibited a comparable distribution of internalized nanobody across GM130‐, EEA1‐, and LAMP1‐positive compartments. Upon incubation of cells with VHH‐tev‐mCherry under conditions identical to those applied for MGAT2, we observed a similar extent of colocalization with GM130 for most reporters (Fig. 5A–E and Fig. S4A). A notable exception was ManII, for which nanobody internalization resulted in only moderate overlap with GM130‐positive structures (Fig. 5C). The rather low degree of colocalization of ManII with GM130 might also explain the relatively weak sulfation of internalized VHH‐tev‐4xTS compared to the other reporters (Fig. 3F). Consistent with our observations for MGAT2, a substantial fraction of nanobody associated with these other Golgi enzyme reporters was detected in LAMP1‐positive compartments, indicative of lysosomal localization (Fig. 5F–H). These findings suggest that Golgi enzymes internalized from the cell surface are retrieved to the TGN but also directed from EEA1‐positive early endosomes toward the endolysosomal pathway.

**Fig. 5:**
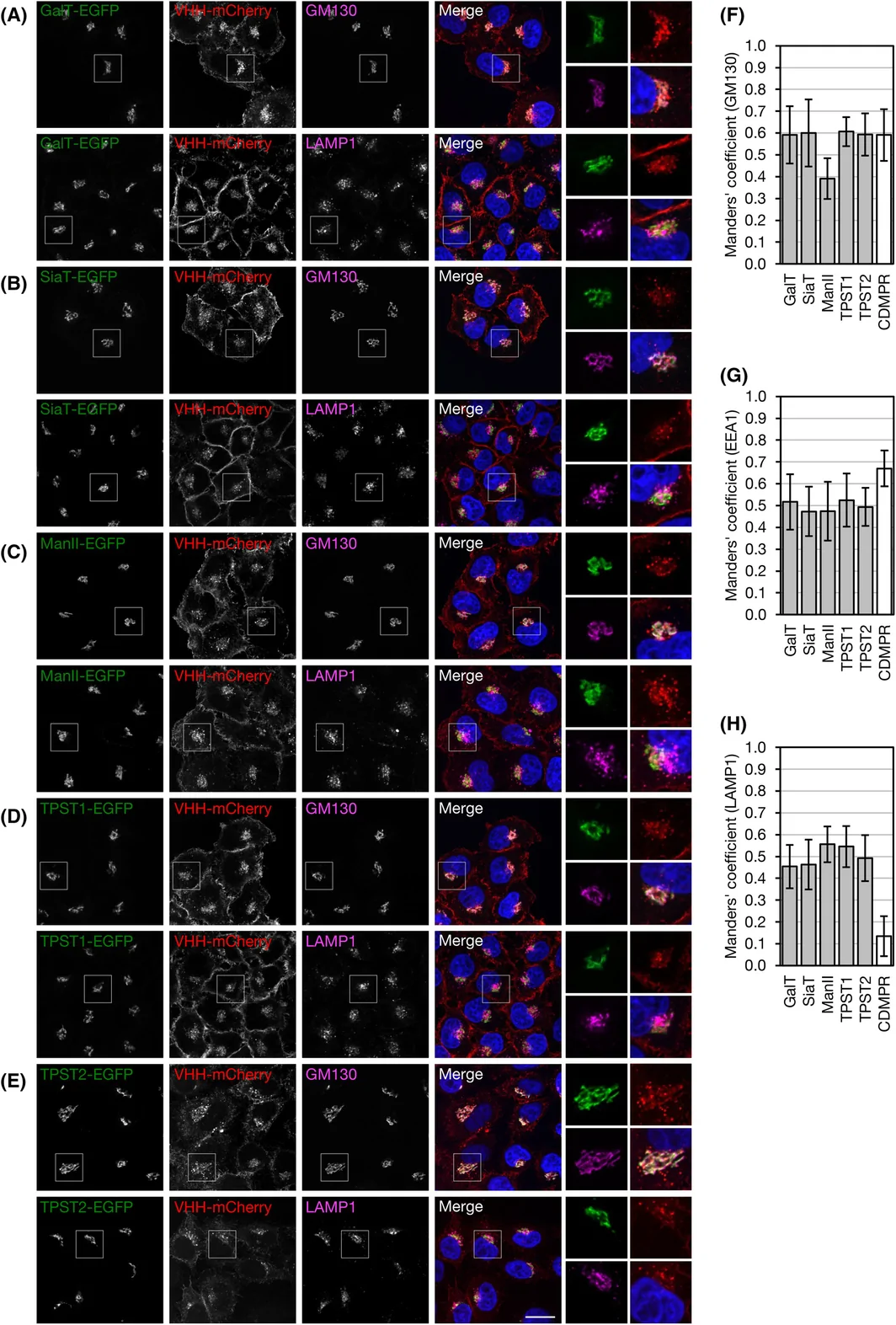
Quantitative compartment colocalization analysis of Golgi enzyme‐mediated uptake of fluorescent nanobody. (A–E) HeLa cells stably expressing the indicated EGFP‐tagged Golgi enzymes were allowed to endocytose VHH‐tev‐mCherry (at 5 μg·mL^−1^) for 5 h, fixed, stained for GM130 or LAMP1, and nuclei (DAPI, blue), and imaged by fluorescence microscopy. Insets display enlarged regions of the selected area. For figure preparation, brightness was adjusted for visualization only. When applicable, all quantitative analyses were performed using the original, unprocessed image data. Bar: 20 μm. (F– H) Quantitative compartment colocalization analysis of EGFP‐tagged GalT, SiaT, ManII, TPST1, and TPST2 with GM130 (Golgi), EEA1 (early endosomes), or LAMP1 (lysosomes). Manders' correlation coefficients (‘compartment signal’ in ‘mCherry signal’) were determined to quantify the extent of colocalization between EGFP‐tagged reporters and GM130, EEA1, or LAMP1. Manders' correlation coefficients of CDMPR are included as a quantitative reference (white bar derived from Fig. 4). For each condition, 50–60 cells from randomly selected Z‐stack acquisitions were evaluated across three independent experiments. Data represent mean values ± standard deviation from three biological replicates.

To exclude the possibility that amino acid sequences within the lumenal domains—absent in our truncated GalT, SiaT, and ManII reporter constructs—might contribute to the prevention of endosome‐to‐Golgi retrieval, we employed full‐length (FL) versions of these enzymes. Similar to their truncated counterparts, transiently expressed full‐length GFP‐tagged reporters facilitated nanobody delivery to the sulfation‐competent compartment (Fig. S5A,C). Golgi retrieval of recombinant GalT(FL), SiaT(FL), and ManII(FL) was further substantiated by fluorescence microscopy (Fig. S5A,B). For GalT, we additionally examined whether differences in the cytoplasmic domain might influence cell surface appearance and subsequent nanobody‐mediated transport to the TGN. While both the short and long GalT isoforms share an 11‐amino acid cytoplasmic tail, the long variant contains an additional 13‐amino acid extension. As demonstrated by sulfation assays and microscopy, the short GalT (shGalT) similarly trafficked to post‐Golgi compartments, bound nanobodies at the cell surface, and facilitated their piggyback transport to the sulfation compartment (Fig. S4B,D).

Notably, several additional Golgi enzymes—including MGAT1, B4GalT7, and Man1B1—also exhibited a detectable cell surface pool, as inferred from nanobody accessibility (Fig. S6A,C). Nanobody uptake by these reporters frequently coincided with the presence of mCherry signal within or close to GM130‐positive compartments (Fig. S6B). To further validate TGN arrival biochemically, we selected B4GalT7‐EGFP and assessed nanobody delivery in a transient transfection assay (Fig. S6D). B4GalT7‐EGFP underwent efficient TGN retrieval upon nanobody internalization, as evidenced by the appearance of radioactively labeled VHH‐tev‐4xTS. This observation is consistent with previous findings [12] where TGN delivery of internalized anti‐myc antibodies bound to myc‐tagged B4GalT7 was demonstrated. Whether other reporters examined in that study follow a similar pattern of nanobody sulfation remains to be determined. While many Golgi enzymes were capable of reaching the cell surface and undergoing retrieval, FucT‐EGFP represented an exception, exhibiting no detectable surface localization and, consequently, no nanobody uptake or delivery to the TGN (Fig. S6A–D). In this regard, FucT thus serves as an additional negative control for our EGFP fusion reporters. In general, nanobody internalization was observed only when the EGFP moiety was presented extracellularly by the reporter construct. Cells stably expressing cytosolic EGFP did not (Fig. S4C).

### Golgi‐IP demonstrates retrieval of Golgi enzymes from the plasma membrane

To further substantiate our observations obtained by radioactive sulfation assays (Fig. 3) and fluorescence microscopy (Figs 4 and 5), we sought to determine whether internalized nanobodies could also be detected biochemically within Golgi‐positive membranes through selective isolation of intracellular compartments. Robust and highly purified enrichment procedures for the Golgi apparatus, as well as other organelles—including lysosomes and early endosomes—have previously been established for biochemical, lipidomic, and proteomic analyses. Isolation of these compartments is commonly achieved by tagging bona fide resident proteins with high‐affinity epitopes, such as HA or FLAG, followed by their expression in the target cells [34, 35, 36]. Given our observations that internalized nanobodies accumulated within both Golgi and LAMP1‐positive compartments, we aimed to assess the presence of the protein binder in these organelles using Golgi‐IP and Lyso‐IP approaches. To this end, we generated stable HeLa cell lines expressing the reporter proteins TMEM115‐3xHA (GolgiTAG) or TMEM192‐3xHA (LysoTAG) and verified their expression and defined subcellular localization. TMEM115‐3xHA was readily detected and exhibited strong colocalization with the Golgi marker Giantin (Fig. 6A,B), whereas TMEM192‐3xHA was largely excluded from Giantin‐positive structures (Fig. 6A,B). These observations are fully consistent with previous studies that established and extensively characterized the GolgiTAG and LysoTAG reporter systems [34, 35].

**Fig. 6:**
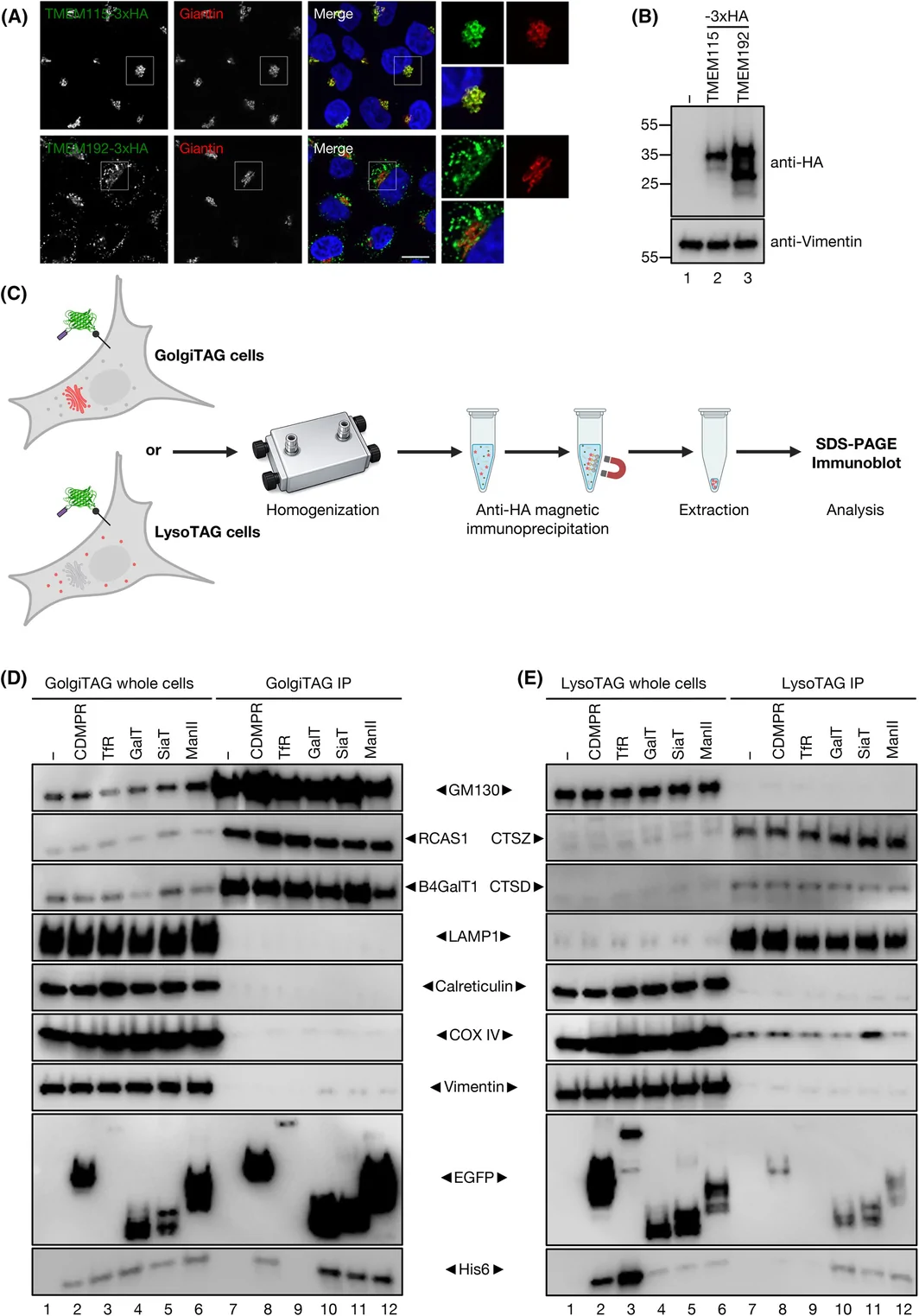
Golgi‐IP and Lyso‐IP methods reveal compartment‐delivered nanobody by Golgi enzymes from the cell surface. (A) HeLa cells stably expressing TMEM115‐3xHA or TMEM192‐3xHA were fixed, stained for HA, Giantin, and nuclei (DAPI, blue), and imaged by fluorescence microscopy. Insets display enlarged regions of the selected area. For figure preparation, brightness was adjusted for visualization only. When applicable, all quantitative analyses were performed using the original, unprocessed image data. Bar: 20 μm. (B) HeLa cells stably expressing TMEM115‐3xHA or TMEM192‐3xHA were analyzed by SDS‐gel electrophoresis and immunoblotting using anti‐HA and ‐vimentin antibodies. While the GolgiTAG reporter runs as a single band, the LysoTAG reporter runs as two discrete bands. The positions of size markers with molecular mass in kiloDaltons are indicated. (C) Graphical demonstration of the Golgi‐IP and Lyso‐IP workflow. GolgiTAG and LysoTAG cells were transiently transfected with EGFP reporters, followed by incubation with nanobody (VHH‐tev‐4xTS). Cells were homogenized and cleared supernatant containing intracellular organelles was incubated with magnetic anti‐HA beads. Beads were washed and subjected to SDS/PAGE and immunoblot analysis. The icon composed of a black line connected to folded EGFP represents transfected EGFP reporter, the violet rectangular‐like shape represented nanobody captured on the cell surface. Figure was created with BioRender.com. (D) Immunoblotting for protein markers of various subcellular compartments in whole cell lysates and purified Golgi using Golgi‐IP. The Golgi‐IP isolates pure Golgi membranes. GolgiTAG cells were transfected with plasmids encoding EGFP fusions of CDMPR, TfR, GalT, SiaT, and ManII, followed by uptake with nanobody (VHH‐tev‐4xTS). Untransfected GolgiTAG cells incubated with nanobody served as control. Whole cell lysates as well as the resuspended immunoprecipitates (IPs) were subjected to immunoblotting with the indicated compartment‐specific antibodies, among them are three Golgi‐specific markers: GM130, RCAS1, and B4GalT1. Anti‐His6 detects VHH‐tev‐4xTS in whole cell lysates or IP. (E) Immunoblotting for protein markers of various subcellular compartments in whole cell lysates and purified lysosomes using Lyso‐IP. The Lyso‐IP isolates pure lysosomes. LysoTAG cells were transfected with plasmids encoding EGFP fusions of CDMPR, TfR, GalT, SiaT, and ManII, followed by uptake with nanobody (VHH‐tev‐4xTS). Untransfected LysoTAG cells incubated with nanobody served as control. Whole cell lysates as well as the resuspended immunoprecipitates (IPs) were subjected to immunoblotting with the indicated compartment‐specific antibodies, among them are three lysosome‐specific markers: LAMP1, CTSD, and CTSZ. Anti‐His6 detects VHH‐tev‐4xTS in whole cell lysates or IP.

Having confirmed the proper localization of the GolgiTAG and LysoTAG constructs in HeLa cells, we next transiently expressed EGFP‐tagged CDMPR, TfR, and our battery of Golgi enzymes in these cells, followed by internalization of VHH‐tev‐4xTS for 5 h at 37 °C. Cells were subsequently homogenized, and Golgi and lysosomal membranes were isolated by anti‐HA immunoprecipitation using magnetic beads according to previously described procedures (Fig. 6C) [34].

Immunoblot analysis of the Golgi‐IP bead‐bound fraction revealed a pronounced enrichment of endogenous Golgi proteins, including GM130, RCAS1, and β‐1,4‐galactosyltransferase 1, whereas markers of lysosomes (LAMP1), the ER (calreticulin), mitochondria (COX IV), and the cytosol (vimentin) were markedly depleted (Fig. 6D). Golgi‐IP fractions were thus essentially devoid of protein contaminants originating from other secretory or endocytic compartments. Similar to endogenous Golgi markers, EGFP‐tagged GalT, SiaT, ManII, TPST1, TPST2, and MGAT2 were also robustly enriched in the Golgi‐IP fraction, demonstrating their predominant localization to the Golgi complex under steady‐state conditions (Fig. 6D and Fig. S7A). As expected, recombinant CDMPR was likewise strongly enriched in Golgi‐IP fractions, whereas TfR was detectable but not substantially enriched. While MPRs continuously cycle between the TGN and endosomes as part of their physiological trafficking itinerary, TfR primarily traverses the Golgi en route to early endosomes and the plasma membrane (Fig. 6D). Although nanobody (detected with anti‐His6) was detectable in homogenates of all transfected cells owing to surface binding and subsequent internalization, VHH‐tev‐4xTS was exclusively detected in Golgi‐IP fractions derived from cells expressing CDMPR and Golgi enzymes, reflecting nanobody delivery to the Golgi lumen via retrograde transport (Fig. 6D and Fig. S7A). Nanobody internalized by TfR was not detected in Golgi‐IP fractions, consistent with our sulfation‐based readout (Fig. 3D) and previous reports [16]. This Golgi‐IP assay fully corroborates our observations of Golgi enzyme retrieval obtained by sulfation analysis and microscopy.

Since nanobody localization following reporter‐mediated internalization was not exclusively restricted to GM130‐positive compartments but also overlapped with LAMP1‐positive structures (Figs 4 and 5), we employed Lyso‐IP to assess Golgi enzyme–nanobody trafficking from the cell surface to lysosomes (Fig. 6E and Fig. S7B). Lyso‐IP fractions exhibited a pronounced enrichment of lysosomal proteins (LAMP1, cathepsin Z, and cathepsin D), whereas markers of other cellular compartments (GM130, calreticulin, COX IV, and vimentin) were substantially depleted or absent. Only trace amounts of EGFP‐tagged CDMPR, TfR, and Golgi enzymes were detected using anti‐GFP antibodies in Lyso‐IP samples relative to their corresponding homogenates. This is consistent with their steady‐state distribution, as these proteins are not predominantly localized to lysosomes. We also detected lumenal nanobody in Lyso‐IP fractions from cells expressing the Golgi enzyme reporters, suggesting that internalized nanobody is not only delivered to the Golgi but also accumulates within lysosomes. Collectively, our findings demonstrate that a fraction of internalized nanobody undergoes retrieval to the TGN, whereas another fraction localizes to LAMP1‐positive compartments.

To what extent the detected surface expression of the investigated Golgi enzymes reflects their physiological distribution remains to be determined. We cannot exclude that the observed level of reporter surface abundance represents an experimental artifact, as the plasma membrane frequently serves as a default destination for overexpressed secretory cargo. For this reason, we sought to generate stable cell lines exhibiting more moderate and homogeneous expression of the recombinant GFP fusion proteins. Tracing endogenous Golgi enzymes or even detecting their low cell surface abundance by proteomic approaches is aspirational but may prove challenging, as the copy number of these proteins per cell is in general relatively low. For example, TPST2 is present at approximately 600 copies per cell and MGAT2 at roughly 30 000 copies per cell, in striking contrast to TfR, which is expressed at more than 5 million copies per cell [37]. Nevertheless, the observation that a fraction of the Golgi enzymes, but not TfR, H1, or LAMP1, relocalizes to the Golgi following surface exposure is unlikely to represent a stochastic event and instead strongly suggests the existence of a machinery‐driven retrieval pathway.

While a comprehensive screen to identify the molecular machinery governing endosome‐to‐Golgi retrieval lies beyond the scope of the present study, existing evidence suggests a potential role for AP‐5 in the retrieval of select Golgi‐resident proteins [13]. In that study, GOLM1 and GALNT2 were among the most prominently depleted proteins in vesicle‐enriched fractions derived from adaptor protein (AP)‐5 complex knockout cells [13]. As a starting point for further investigation along these lines, we examined whether GOLM1‐EGFP and GALNT2‐EGFP undergo cycling to the cell surface followed by retrograde retrieval to the TGN. Strikingly, nanobodies internalized via recombinant GOLM1 or GALNT2 were sulfated and localized to GM130‐positive membranes (Fig. S8A–C). Whether AP‐5 directly regulates their endosome‐to‐Golgi transport remains to be elucidated.

Our findings provide direct experimental support for a previously underappreciated recycling route of Golgi enzymes to the TGN and help reconcile earlier observations suggesting predominant retention with emerging evidence for dynamic retrieval. Furthermore, our data confirm that Golgi enzymes also partially engage the endolysosomal pathway following endocytosis as reported elsewhere [12]. It has to be mentioned, though, that a few Golgi enzymes—MGAT2, B3GalT6, B4GalT7, and POMGNT1—were likewise reported to retrieve to the TGN in the study by the Lu laboratory [12].

We have not directly examined whether internalized Golgi enzymes detected within LAMP1‐positive compartments are indeed destined for degradation. An alternative interpretation of Golgi enzyme colocalization with LAMP1‐positive structures is that retrograde transport may originate from late endosomal or lysosomal membranes. Consistent with this notion, AP‐5 has been shown to predominantly localize to LAMP1‐decorated compartments rather than to EEA1‐positive early endosomes, where it mediates late endosome‐to‐Golgi retrieval. Recruitment of the AP‐5/SPG11/SPG15 complex has been demonstrated to depend on Rag GTPases and phosphatidylinositol‐3‐phosphate (PI3P) [38]. Using our sulfation assay, it may therefore be of considerable interest to investigate whether AP‐5 contributes to the retrieval of additional Golgi enzymes beyond those already validated [13], including the candidates examined in the present study. Furthermore, advanced methodologies such as SPEx could facilitate the characterization of the proteomic composition of reporter–nanobody–LAMP1‐positive structures, thereby providing deeper insights into their molecular identity and functional organization [39].

The potential trafficking routes of Golgi enzymes that transiently reside at the cell surface are summarized schematically (Fig. 7). Enzymes persisting at the plasma membrane may exert noncanonical functions, such as acting as adhesion molecules through interactions with extracellular ligands or may be released into the extracellular milieu via ectodomain shedding as soluble enzymes [11, 40]. Alternatively, once internalized, Golgi enzymes enter the endosomal system, from which they can either be retrieved to the TGN or targeted to lysosomes.

**Fig. 7:**
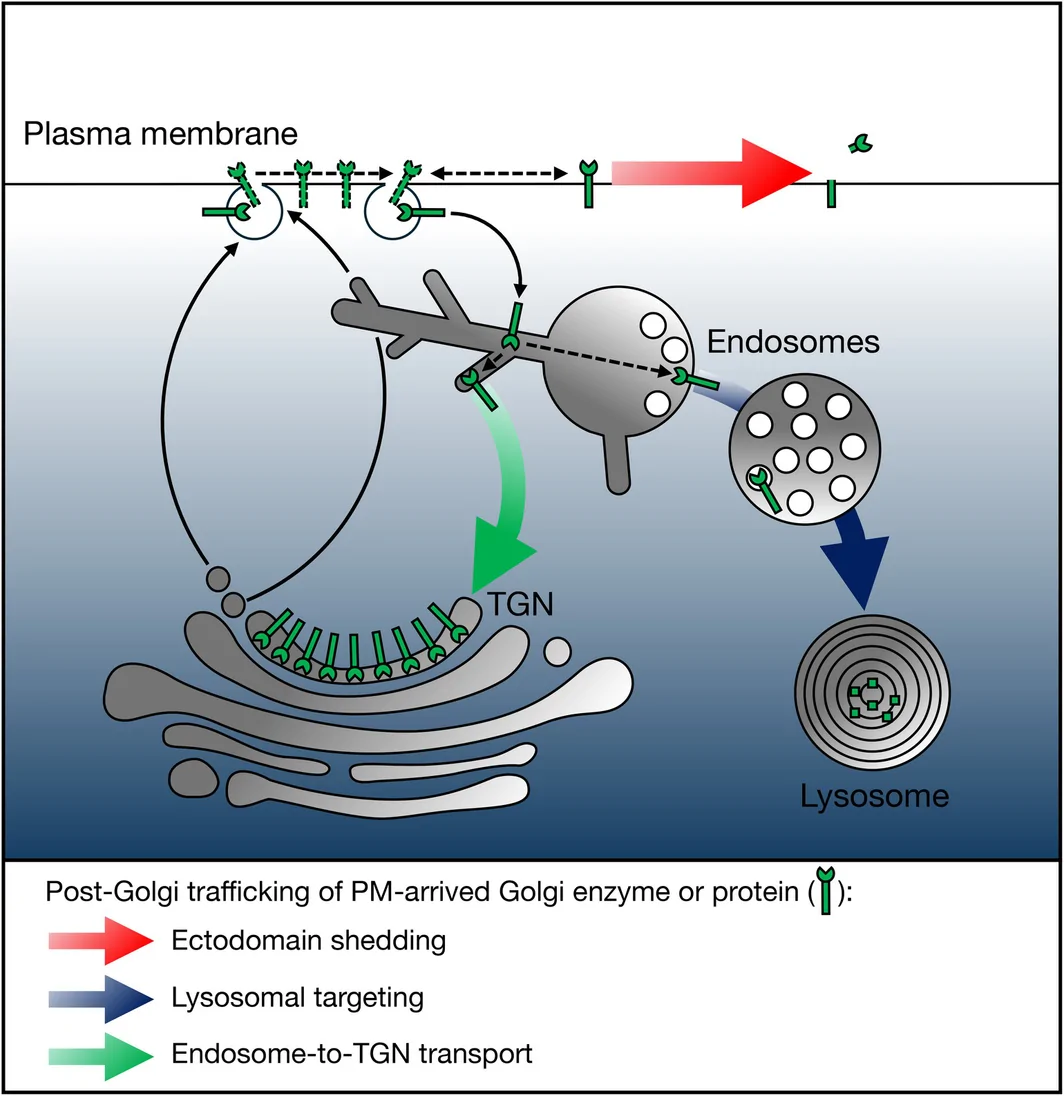
Trafficking itineraries of Golgi enzymes upon arrival at the cell surface: interaction, shedding, or endocytosis. Surface‐residing Golgi enzyme (green cargo) can in principle follow three different routes. When not operating as adhesion Golgi enzymes present at the cell surface (green cargo), they can, in principle, follow three distinct trafficking routes. In the absence of functional engagement as adhesion molecules through interactions with extracellular ligands, their luminal domains may be released into the extracellular milieu via proteolytic cleavage through ectodomain shedding (red arrow). Alternatively, Golgi enzymes may undergo endocytosis and be delivered to early endosomal compartments. From there, sorting determinants govern whether these proteins are directed along the endolysosomal pathway (blue arrow) or rerouted to the trans‐Golgi network (TGN) (green arrow). As the molecular machinery and precise intracellular origin of endosome‐to‐Golgi retrieval for Golgi enzymes remain unresolved, early and late endosomal compartments are collectively denoted as endosomes. Accordingly, the green arrow indicating Golgi retrieval should be seen as originating from an endolysosomal compartment rather than only early endosomes.

In the present study, we demonstrated by three independent approaches—sulfation assays, fluorescence imaging, and Golgi‐IP—that a fraction of surface‐exposed Golgi enzymes undergoes retrieval to the Golgi complex. Our work thus opens new avenues for dissecting the molecular determinants and pathways governing retrograde trafficking of Golgi‐resident proteins.

## Materials and methods

### Bacterial expression and purification of functionalized nanobodies

Functionalized nanobodies were bacterially expressed and isolated as previously described [16, 41]. Briefly, bacterial expression vectors encoding derivatized VHH nanobodies and myc‐BirA (#109424; Addgene, Watertown, MA, USA) were transformed together into BL21 (DE3) competent *E. coli* cells (New England Biolabs, Ipswich, MA, USA), and plated on LB plates with 50 μg·mL^−1^ kanamycin and 50 μg·mL^−1^ carbenicillin. A 20‐mL overnight culture of a single colony was diluted into 1 L LB medium with antibiotics and 200 μm d‐biotin and grown to an OD_600_ of 0.6–0.7 at 37 °C. Expression was induced with 1 mm isopropyl‐β‐*d*‐thiogalactopyranoside (IPTG) at 30 °C for 4 h (VHH‐tev‐std, VHH‐tev‐4xTS) or at 16 °C overnight (VHH‐tev‐mCherry). Cells were pelleted at 5000× ***g*** at 4 °C for 45 min and stored at −80 °C. Upon thawing, they were resuspended in 30 mL PBS with 20 mm imidazole, 200 μg·mL^−1^ lysozyme, 20 μg·mL^−1^ DNase I, 1 mm MgCl_2_, and 1 mm PMSF, incubated for 10 min at room temperature and 1 h at 4 °C while rotating, followed by mechanical lysis using a tip sonicator for 3 times 30 s with 1‐min cooling periods. The lysate was cleared by centrifugation at 15 000× ***g*** for 1 h at 4 °C and loaded on a His GraviTrap column (Cytiva, Marlborough, MA, USA), washed with 20 mm imidazole in PBS, and eluted with 2 mL PBS with 500 mm imidazole. The purified nanobodies were desalted on PD‐10 columns (Cytiva), concentrated to 2 mg·mL^−1^, flash‐frozen in liquid nitrogen, and stored at −80 °C. Plasmids for nanobody fusion protein expression are deposited with Addgene upon publication (#109423, VHH‐tev‐std; #247150, VHH‐tev‐4xTS; #117752, VHH‐tev‐mCherry). Typically, 2 μg·mL^−1^ purified VHH‐tev‐std or VHH‐tev‐4xTS, or 5 μg·mL^−1^ purified VHH‐tev‐mCherry was administered to cells.

### Cell culture, retroviral transduction, and plasmids

HeLa α cell lines (RRID:CVCL_1922) were maintained either in high‐glucose Dulbecco's modified Eagle's medium (DMEM) supplemented with 2 mm L‐glutamine or in high‐glucose DMEM with GlutaMAX‐I as basal medium source. Basal medium was further complemented with 10% fetal calf or bovine serum (FCS or FBS), 100 units/mL streptomycin, and appropriate selection antibiotics (1.5 μg·mL^−1^ puromycin) for proper maintenance of the transduced transgene. The HeLa α cell line used in this study was a kind gift from Prof. em. Martin Spiess. The cell line was authenticated and proven to be human using a commercial cell line authentication service (Microsynth, Balgach, Switzerland) [26]. Phoenix Ampho packaging cells (from the Nolan laboratory, Stanford University, Stanford, CA, USA, RRID:CVCL_H716) were grown in complete medium supplemented with 1 mm sodium pyruvate. Both HeLa α and Phoenix Ampho packaging cells were grown and maintained at 37 °C in 5.0–7.5% CO_2_. All cell lines used in this study were routinely tested for mycoplasma contamination and were confirmed to be mycoplasma‐free.

HeLa cells stably expressing EGFP‐CDMPR, EGFP‐TGN46, TfR‐EGFP, GalT‐EGFP, TPST1‐EGFP, TPST2‐EGFP, and EGFP‐CNX were previously described [16, 41, 42].

To generate HeLa cells stably expressing ManII‐EGFP, SiaT‐EGFP, EGFP‐ERGIC53, EGFP‐LAMP1, H1‐EGFP, shGalT‐EGFP, EGFP‐CD59, EGFP‐FR1, EGFP‐CD8‐Furin, MGAT2‐EGFP, EGFP, TMEM115‐3xHA, or TMEM192‐3xHA, Phoenix Ampho packaging cells were transfected with pQCXIP‐ManII‐EGFP, pQCXIP‐SiaT‐EGFP, pQCXIP‐EGFP‐ERGIC53, pQCXIP‐EGFP‐LAMP1, pQCXIP‐H1‐EGFP, pQCXIP‐shGalT‐EGFP, pQCXIP‐EGFP‐CD59, pQCXIP‐EGFP‐FR1, pQCXIP‐EGFP‐CD8‐Furin, pQCXIP‐MGAT2‐EGFP, pQCXIP‐EGFP, pQCXIP‐TMEM115‐3xHA, or pQCXIP‐TMEM192‐3xHA using FuGENE HD (Promega). The viral supernatant was harvested after 48 h, passed through a 0.45 μm filter, supplemented with 15 μg·mL^−1^ polybrene, and added to target HeLa α cells. The next day, complete medium with 1.5 μg·mL^−1^ puromycin was added for selection, and pooled resistant clones were used for experiments. All EGFP reporter cell lines were further subjected to cell sorting on a FACSAria III (BD Biosciences) to obtain single clones or a cell pool with homogeneous expression levels.

Where indicated, transient transfection of EGFP reporters was performed using FuGENE HD according to the manufacturer's instructions. Cells were harvested for lysis or fixed 24 h after transfection.

For western blot analysis, cells were harvested in lysis buffer (1% Triton X‐100 and 0.5% sodium deoxycholate in PBS) supplemented with 2 mm PMSF and protease inhibitor cocktail (cOmplete, mini EDTA‐free, Sigma‐Aldrich 11836170001, St. Louis, MO, USA) and postnuclear supernatant was prepared following a 15‐min spin at 10 000× ***g*** at 4 °C.

If not stated differently or referred to other studies, all plasmids have been cloned for this study. Coding sequences of interest were amplified by PCR using gene‐specific primers containing appropriate restriction sites. PCR products and the destination vectors (pQCXIP or pEGFP‐N3; Takara Bio Europe, Saint‐Germain‐en‐Laye, France) were digested with the restriction enzymes (New England Biolabs) and ligated using T4 DNA ligase (New England Biolabs) according to the manufacturer's instructions. Ligation mixtures were transformed into chemically competent NEB 5‐alpha *Escherichia coli* cells (New England Biolabs) and transformants were selected on LB agar supplemented with the appropriate antibiotic. Plasmid DNA was isolated from individual colonies and screened by restriction analysis. All constructs were verified by Sanger sequencing (Microsynth) to confirm the presence and correct orientation of the inserts.

cDNA sequences of GalT, SiaT, and ManII were generous gifts from Jennifer Lippincott‐Schwartz (Howard Hughes Medical Institute's Janelia Research Campus, USA). To generate ERGIC53 and LAMP1 constructs, plasmids encoding LMAN1‐bio‐His (Addgene: #51922) or LAMP1‐mGFP (Addgene: #34831) were kind gifts from Gavin Wright or Esteban Dell'Angelica, respectively. Plasmids encoding full‐length sialyltransferase (ST6Gal1) fused to GFP termed ST‐GFP (Addgene: #162500), full‐length mannosidase alpha class 2A member 1 (Man2a1) fused to GFP termed Man2a1‐GFP (Addgene: #163649), and full‐length mannosidase alpha class 1B member 1 (Man1B1) fused to GFP termed Man1B1‐GFP (Addgene: #163646) were kind gifts from Lei Lu. To generate a plasmid conferring Golgi‐IP reporter expression from pQCXIP, a construct expressing TMEM115‐3xHA (DU68534) was purchased from MRCPPU (University of Dundee). To subclone the Lyso‐IP reporter gene into pQCXIP, a plasmid encoding TMEM192‐3xHA was kindly used and provided by David Sabatini (Addgene: #102930). A plasmid encoding a truncated, catalytic domain‐free version of fucosyltransferase 6 (FUT6) modified with GFP named FucT‐EGFP was a kind gift from Jack Rohrer (ZHAW, Switzerland). cDNAs encoding CD59 or folate receptor 1 were obtained from Martin Spiess (Biozentrum, Switzerland). A vector encoding CD8‐Furin was a generous gift by Matthew Seaman (CIMR, UK). cDNA sequences encoding MGAT1, MGAT2, GOLM1, GALNT2, and B4GalT7 were commercially synthesized by Integrated DNA Technologies or Twist Biosciences (both USA). A plasmid containing cDNA with full‐length galactosyltransferase (B4GalT1 #HG11220‐UT) was purchased from Sino Biological Europe, Eschborn, Germany.

Generated reporter plasmids will be deposited with Addgene upon publication (#182642, pQCXIP‐EGFP‐CDMPR; #247140, pQCXIP‐EGFP‐TGN46; #243763, pQCXIP‐TfR‐EGFP; #247141, pQCXIP‐EGFP‐CNX; #247142, pQCXIP‐EGFP‐ERGIC53; #247143, pQCXIP‐EGFP‐LAMP1; #247144, pQCXIP‐H1‐EGFP; #247145, pQCXIP‐GalT‐EGFP; #247146, pQCXIP‐SiaT‐EGFP; #247147, pQCXIP‐ManII‐EGFP; #247148, pQCXIP‐TPST1‐EGFP; #247149, pQCXIP‐TPST2‐EGFP; #254482, pQCXIP‐MGAT1‐EGFP; #254483, pQCXIP‐MGAT2‐EGFP; #254484, pQCXIP‐shGalT‐EGFP; #254485, pQCXIP‐B4GalT7‐EGFP; #254486, pQCXIP‐GALNT2‐EGFP; #254487, pQCXIP‐EGFP‐CD59; #254488, pQCXIP‐EGFP‐FR1; #254489, pQCXIP‐EGFP‐CD8‐Furin; #254490, pGOLM1‐EGFP; #254491, pGalT(FL)‐EGFP; #254492, pQCXIP‐EGFP; #258542, pQCXIP‐TMEM115‐3xHA; #258543, pQCXIP‐TMEM192‐3xHA).

### Gel electrophoresis, immunoblot analysis, and Coomassie staining

Proteins separated by SDS‐gel electrophoresis (7.5–15% polyacrylamide) were transferred to Immobilon‐P^SQ^ PVDF membranes (Millipore). Alternatively, proteins were size‐separated using Mini‐PROTEAN TGX gels (#4561093 and #4561096; Bio‐Rad, Hercules, CA, USA) and transferred using Trans‐Blot Turbo Mini PVDF transfer packs (#1704156; Bio‐Rad). Membranes were blocked with 5% nonfat dry milk in TBS (50 mm Tris/HCl, pH 7.6, 150 mm NaCl) containing 0.1% Tween‐20 (TBST) for 1 h and incubated with primary antibodies diluted in 1% BSA in TBST overnight at 4 °C. After washing, membranes were incubated with HRP‐conjugated secondary antibodies in 1% BSA in TBST for 1 h at room temperature. Signals were detected using Immobilon Western Chemiluminescent HRP Substrate (#WBKLS0500; Millipore) and imaged using a Vilber Fusion Absolute imaging system. Images were processed and assembled using fiji.

For in‐gel staining of purified nanobodies, gels were stained with Coomassie Brilliant Blue R‐250 staining solution (#1610436; Bio‐Rad) for 30 min at room temperature, followed by several washes with Coomassie Brilliant Blue R‐250 destaining solution (#1610438; Bio‐Rad) until protein bands were clearly visible.

### Fluorescence microscopy and image analysis

For immunofluorescence staining, cells were grown on 18‐mm glass coverslips (No. 1.5H), fixed with 3% paraformaldehyde (PFA) for 10 min at room temperature, washed with PBS, quenched with 50 mm NH_4_Cl in PBS for 5 min, permeabilized with 0.1% Triton X‐100 in PBS for 10 min, blocked with 1% BSA in PBS for at least 15 min, incubated with primary antibody in 1%BSA/PBS for 2 h, washed, and stained with fluorescent secondary antibodies in 1% BSA/PBS for 1 h. After a 5 min‐stain with 5 μg·mL^−1^ DAPI and three washes with PBS, coverslips were mounted in Fluoromount‐G (#0100‐01; Southern Biotech, Birmingham, AL, USA) or ProLong Diamond antifade (#P36965; Thermo Fisher Scientific, Waltham, MA, USA). Staining patterns of Z‐stacks were acquired using a Zeiss LSM700 point‐scanning confocal microscope or a Nikon Ti2 microscope equipped with a Crest X‐Light V3 spinning disk confocal system.

For colocalization image analysis, confocal fluorescence images were deconvolved using huygens professional (version 25.10) with a theoretical point spread function (PSF) using the automatic algorithm and a conservative deconvolution setting. Rectangular regions of interest (ROIs) were defined on deconvolved images, and Pearson's and thresholded Manders' colocalization coefficients were calculated using the JACoP plugin in fiji. For subsequent analysis, the biologically more informative thresholded Manders' coefficient, reflecting the fraction of the respective compartment signal overlapping with mCherry signal (‘compartment signal’ in ‘mCherry signal’) was used.

### Sulfation analysis

To analyze retrograde transport to the compartment of sulfation, cells were incubated with 1 mL sulfate‐free medium for 1 h at 37 °C and 7.5% CO_2_ before labeling with sulfate‐free medium supplemented with 0.5 mCi·mL^−1^ sodium [^35^S]sulfate (Hartmann Analytics S‐RA‐2) and 2 μg·mL^−1^ purified VHH‐tev‐std or VHH‐tev‐4xTS for 1–5 h. Sulfate‐free medium was prepared as described previously [17].

After incubation with radioactive sulfate, cells were washed twice with ice‐cold PBS, lysed in 1 mL lysis buffer containing 2 mm PMSF and protease inhibitor cocktail (cOmplete, mini EDTA‐free) and centrifuged at 10 000× ***g*** for 15 min at 4 °C. A fraction (50–100 μL) of the postnuclear supernatants was saved and used for immunoblot analysis of total cell‐associated nanobody and as an actin control. The rest was incubated for 1 h at 4 °C with 20 μL Ni Sepharose High Performance beads (GE Healthcare Life Sciences) to isolate the nanobodies. Beads were washed three times with lysis buffer containing 20 mm imidazole and boiled in SDS‐sample buffer, followed by bead removal using MiniSpin columns (NanoTag Biotechnologies A1001‐L, Göttingen, Germany). Isolated nanobodies were analyzed by SDS‐gel electrophoresis and autoradiography using Storage Phosphor Screens and a Typhoon FLA7000 IP phosphorimager (GE Healthcare Life Sciences, Uppsala, Sweden) or Amersham Typhoon IP phosphorimager (Cytiva).

### Golgi‐IP and Lyso‐IP

To isolate Golgi or lysosomal membranes, a method and protocol as previously established was adapted and applied [34, 35]. Briefly, cells stably expressing TMEM115‐3xHA (for Golgi‐IP) or TMEM192‐3xHA (for Lyso‐IP) were grown in large 15 cm diameter dishes and transiently transfected with 20 μg EGFP reporter plasmid DNA and 60 μL using FuGENE HD. About 24–36 h post‐transfection, cells were incubated with VHH‐tev‐4xTS for 5 h at 37 °C to allow nanobody internalization. Following two washes with PBS and one wash with KPBS, transfected and nanobody‐labeled Golgi‐ and Lyso‐IP reporter cells were harvested in 1.5 mL of ice‐cold KPBS supplemented with 2 mm PMSF, protease inhibitor cocktail (cOmplete, mini EDTA‐free), and PhosSTOP (4906837001 Sigma‐Aldrich). After gentle pelleting of scraped cells at 1000× ***g*** for 2 min at 4 °C, cells were resuspended in 1.2 mL supplemented KPBS and homogenized with a ball‐bearing cell homogenizer (ball clearance 18 μm; Isobiotec, Heidelberg, Germany) connected to 2 mL‐syringes. Homogenization was performed by pressing the sample 25 times through the device. Homogenates were cleared of debris by centrifugation at 1000× ***g*** for 2 min at 4 °C. The supernatant which contains intracellular compartments was collected and transferred to a clean 1.5‐mL Eppendorf tube containing 120 μL of 25% slurry of washed anti‐HA magnetic beads (#88837; Thermo Fisher Scientific). The mixture was then incubated at 4 °C for 25 min on an end‐over‐end rotator. After incubation, the tube containing the mixture was placed on a DynaMag Spin Magnet (#12320D; Thermo Fisher Scientific) for 30 s and the supernatant was removed. The beads were washed 5 times with cold KPBS and then resuspended in sample buffer and further processed for SDS/PAGE and western blotting.

### Electron microscopy

For immunoelectron microscopy, the procedure as previously outlined was performed [16]. Briefly, EGFP‐modified reporter cell lines were seeded and grown in 100 mm culture dishes. Cells were washed twice with PBS, fixed in 3% formaldehyde and 0.2% glutaraldehyde overnight at 4 °C, scraped, pelleted, resuspended, washed three times in PBS, incubated with 50 mm NH4Cl in PBS for 30 min, washed three times in PBS, dehydrated, and infiltrated with LR‐GOLD resin according to the manufacturer's instructions (London Resin, London, UK), which was allowed to polymerize for 1 day at −10 °C. Sections of 60–70 nm were collected on carbon‐coated Formvar‐Ni‐grids, and then incubated with rabbit anti‐GFP antibodies in PBS, 2% BSA, 0.1% Tween‐20 for 2 h, washed with PBS, and incubated with 10‐nm colloidal gold‐conjugated goat anti‐rabbit immunoglobulin antibodies in PBS, 2% BSA, and 0.1% Tween‐20 for 90 min. Grids were washed five times for 5 min in PBS and five times in H2O, before staining for 10 min in 4% uranyl acetate and 2 min with lead citrate (Reynolds solution). Sections were viewed with a Phillips CM100 electron microscope. Gold particle localization analysis was performed by scoring and quantification of the percentage of gold particles in EM sections of each individual EGFP reporter protein.

### Antibodies

For immunofluorescence microscopy, sheep polyclonal anti‐TGN46 (1 : 1000; #AHP500GT Bio‐Rad), rabbit monoclonal anti‐GM130 (D6B1) (1 : 1000; #12480S Cell Signaling Technology), rabbit monoclonal anti‐EEA1 (C45B10) (1 : 1000; #3288S Cell Signaling Technology), rabbit monoclonal anti‐HA (C29F4) (1 : 2000; #3724S Cell Signaling Technology), rabbit monoclonal anti‐LAMP1 (D2D11) (1 : 1000; #9091S Cell Signaling Technology), and mouse monoclonal anti‐Giantin (made from hybridoma; 1 : 1000) primary antibodies were used. For electron microscopy, rabbit polyclonal anti‐GFP (1 : 500; #ab6556 Abcam, Cambridge, UK) primary antibody was used. For immunoblotting, mouse monoclonal anti‐GFP (1 : 4000;#11814460001 Sigma‐Aldrich), rabbit polyclonal anti‐GFP (1 : 3000; #ab6556 Abcam), mouse monoclonal anti‐actin (1 : 50 000; #MAB1501 Sigma‐Aldrich), rabbit monoclonal anti‐RCAS1 (D2B6N) (1 : 1000; #12290S Cell Signaling Technology), rabbit monoclonal anti‐COX IV (3E11) (1 : 1000; #4850S Cell Signaling Technology), rabbit monoclonal anti‐Calreticulin (D3E6) (1 : 1000; #12238S Cell Signaling Technology), rabbit monoclonal anti‐Vimentin (D21H3) (1 : 1000; #5741S Cell Signaling Technology), rabbit polyclonal anti‐β‐1,4‐Galactosyltransferase 1 (1 : 1000; #NBP1‐88654 Novus Biologicals, Centennial, CO, USA), rabbit polyclonal anti‐Cathepsin Z (1 : 1000; ABC442 Merck Millipore, Burlington, MA, USA), rabbit monoclonal anti‐Cathepsin D (1 : 1000; #ab75852 Abcam), rabbit monoclonal anti‐HA (C29F4) (1 : 3000; #3724S Cell Signaling Technology), mouse monoclonal anti‐HA (made from 12CA5 hybridoma; 1 : 10 000), rabbit polyclonal anti‐His6 (1 : 10 000; #A190‐114A Bethyl Laboratories, Montgomery, TX, USA), and rabbit polyclonal anti‐T7 (1 : 10 000; #A190‐117A Bethyl Laboratories) primary antibodies were used.

As secondary antibodies for immunofluorescence microscopy, Alexa Fluor 568‐labeled donkey anti‐rabbit (1 : 800; #A10042 Thermo Fisher Scientific), Alexa Fluor 647‐labeled donkey anti‐sheep (1 : 800; #A21448 Thermo Fisher Scientific), and Alexa Fluor 647‐labeled donkey anti‐rabbit (1 : 800; #A31573 Thermo Fisher Scientific) immunoglobulin antibodies were used. As secondary antibodies for immunoblotting, HRP‐labeled goat anti‐rabbit (1 : 10 000; #A‐0545 Sigma‐Aldrich) or goat anti‐rabbit (#7074S Cell Signaling Technology) and goat anti‐mouse (1 : 10 000; #A‐0168 Sigma‐Aldrich) or horse anti‐mouse (#7076S Cell Signaling Technology) immunoglobulin antibodies were used.

## Author contributions

DPB contributed to conceptualization, resources, data curation, formal analysis, supervision, validation, investigation, visualization, methodology, project administration, and writing original draft, review, and editing. TJ contributed to formal analysis and investigation.

## Supporting information


**Fig. S1.** Expression of Golgi enzymes and localization of EGFP‐tagged CDMPR, CNX, and ERGIC53.
**Fig. S2.** Expression and localization of additional EGFP reporter proteins.
**Fig. S3.** VHH‐tev‐mCherry for quantitative colocalization of EGFP reporter proteins.
**Fig. S4.** Fluorescent nanobody uptake by reporter and cytosolic tail‐truncated Golgi enzymes.
**Fig. S5.** Expression, localization, and retrograde transport of full‐length recombinant GalT, SiaT, and ManII.
**Fig. S6.** Expression, localization, and retrograde transport of recombinant MGAT2, Man1B1, MGAT1, FucT, and B4GalT7.
**Fig. S7.** Golgi‐IP and Lyso‐IP method reveal compartment‐delivered nanobody by TPST1, TPST2, and MGAT2 from the cell surface.
**Fig. S8.** Expression, localization, and retrograde transport of recombinant GOLM1 and GALNT2.

## Data Availability

All data supporting the conclusions of this article are included within the main article and its Supporting Information. No large‐scale datasets requiring public repository deposition were generated in this study.

## References

[1] Banfield DK (2011) Mechanisms of protein retention in the Golgi. Cold Spring Harb Perspect Biol 3, a005264. doi: 10.1101/cshperspect.a005264 PMC3140682 21525512

[2] Nilsson T , Hoe MH , Slusarewicz P , Rabouille C , Watson R , Hunte F , Watzele G , Berger EG and Warren G (1994) Kin recognition between medial Golgi enzymes in HeLa cells. EMBO J 13, 562–574. doi: 10.1002/j.1460-2075.1994.tb06294.x PMC394845 8313901

[3] Kellokumpu S , Hassinen A and Glumoff T (2016) Glycosyltransferase complexes in eukaryotes: long‐known, prevalent but still unrecognized. Cell Mol Life Sci 73, 305–325. doi: 10.1007/s00018-015-2066-0 PMC7079781 26474840

[4] Tewari R , Bachert C and Linstedt AD (2015) Induced oligomerization targets Golgi proteins for degradation in lysosomes. Mol Biol Cell 26, 4427–4437. doi: 10.1091/mbc.E15-04-0207 PMC4666137 26446839

[5] Bretscher MS and Munro S (1993) Cholesterol and the Golgi apparatus. Science 261, 1280–1281. doi: 10.1126/science.8362242 8362242

[6] Mitra K , Ubarretxena‐Belandia I , Taguchi T , Warren G and Engelman DM (2004) Modulation of the bilayer thickness of exocytic pathway membranes by membrane proteins rather than cholesterol. Proc Natl Acad Sci USA 101, 4083–4088. doi: 10.1073/pnas.0307332101 PMC384699 15016920

[7] Lujan P , Garcia‐Cabau C , Wakana Y , Vera Lillo J , Rodilla‐Ramirez C , Sugiura H , Malhotra V , Salvatella X , Garcia‐Parajo MF and Campelo F (2024) Sorting of secretory proteins at the trans‐Golgi network by human TGN46. elife 12, 91708. doi: 10.7554/eLife.91708 PMC10928510 38466628

[8] Ali MF , Chachadi VB , Petrosyan A and Cheng PW (2012) Golgi phosphoprotein 3 determines cell binding properties under dynamic flow by controlling Golgi localization of core 2 N‐acetylglucosaminyltransferase 1. J Biol Chem 287, 39564–39577. doi: 10.1074/jbc.M112.346528 PMC3501027 23027862

[9] Welch LG , Muschalik N and Munro S (2024) The FAM114A proteins are adaptors for the recycling of Golgi enzymes. J Cell Sci 137, 262160. doi: 10.1242/jcs.262160 PMC11441981 39129673

[10] Taylor RJ , Zubkov N , Ciazynska KA , Kaufman JGG , Tagiltsev G , Owen DJ , Briggs JAG and Munro S (2025) The mechanistic basis of cargo selection during Golgi maturation. Sci Adv 11, eaea0016. doi: 10.1126/sciadv.aea0016 PMC12494035 41042862

[11] Shur BD , Evans S and Lu Q (1998) Cell surface galactosyltransferase: current issues. Glycoconj J 15, 537–548. doi: 10.1023/a:1006951407168 9881758

[12] Sun X , Mahajan D , Chen B , Song Z and Lu L (2021) A quantitative study of the Golgi retention of glycosyltransferases. J Cell Sci 134, 258564. doi: 10.1242/jcs.258564 34533190

[13] Hirst J , Itzhak DN , Antrobus R , Borner GHH and Robinson MS (2018) Role of the AP‐5 adaptor protein complex in late endosome‐to‐Golgi retrieval. PLoS Biol 16, e2004411. doi: 10.1371/journal.pbio.2004411 PMC5806898 29381698

[14] Buser DP and Spang A (2023) Protein sorting from endosomes to the TGN. Front Cell Dev Biol 11, 1140605. doi: 10.3389/fcell.2023.1140605 PMC9988951 36895788

[15] Hirst J , Borner GH , Antrobus R , Peden AA , Hodson NA , Sahlender DA and Robinson MS (2012) Distinct and overlapping roles for AP‐1 and GGAs revealed by the “Knocksideways” system. Curr Biol 22, 1711–1716. doi: 10.1016/j.cub.2012.07.012 PMC3485558 22902756

[16] Buser DP , Schleicher KD , Prescianotto‐Baschong C and Spiess M (2018) A versatile nanobody‐based toolkit to analyze retrograde transport from the cell surface. Proc Natl Acad Sci USA 115, E6227–E6236. doi: 10.1073/pnas.1801865115 PMC6142194 29915061

[17] Buser DP and Spiess M (2019) Analysis of endocytic uptake and retrograde transport to the trans‐Golgi network using functionalized nanobodies in cultured cells. *J Vis Exp*, 10.3791/59111 30855580

[18] Sengupta P , Satpute‐Krishnan P , Seo AY , Burnette DT , Patterson GH and Lippincott‐Schwartz J (2015) ER trapping reveals Golgi enzymes continually revisit the ER through a recycling pathway that controls Golgi organization. Proc Natl Acad Sci USA 112, E6752–E6761. doi: 10.1073/pnas.1520957112 PMC4679030 26598700

[19] Ghosh P , Griffith J , Geuze HJ and Kornfeld S (2003) Mammalian GGAs act together to sort mannose 6‐phosphate receptors. J Cell Biol 163, 755–766. doi: 10.1083/jcb.200308038 PMC2173681 14638859

[20] Nilsson T , Lucocq JM , Mackay D and Warren G (1991) The membrane spanning domain of beta‐1,4‐galactosyltransferase specifies trans Golgi localization. EMBO J 10, 3567–3575. doi: 10.1002/j.1460-2075.1991.tb04923.x PMC453088 1935889

[21] Novikoff PM , Tulsiani DR , Touster O , Yam A and Novikoff AB (1983) Immunocytochemical localization of alpha‐D‐mannosidase II in the Golgi apparatus of rat liver. Proc Natl Acad Sci USA 80, 4364–4368. doi: 10.1073/pnas.80.14.4364 PMC384038 6576342

[22] Rhee SW , Starr T , Forsten‐Williams K and Storrie B (2005) The steady‐state distribution of glycosyltransferases between the Golgi apparatus and the endoplasmic reticulum is approximately 90:10. Traffic 6, 978–990. doi: 10.1111/j.1600-0854.2005.00333.x 16190979

[23] Villeneuve J , Duran J , Scarpa M , Bassaganyas L , Van Galen J and Malhotra V (2017) Golgi enzymes do not cycle through the endoplasmic reticulum during protein secretion or mitosis. Mol Biol Cell 28, 141–151. doi: 10.1091/mbc.E16-08-0560 PMC5221618 27807044

[24] Hathaway HJ , Evans SC , Dubois DH , Foote CI , Elder BH and Shur BD (2003) Mutational analysis of the cytoplasmic domain of beta1,4‐galactosyltransferase I: influence of phosphorylation on cell surface expression. J Cell Sci 116, 4319–4330. doi: 10.1242/jcs.00720 12966167

[25] Newstead S and Barr F (2020) Molecular basis for KDEL‐mediated retrieval of escaped ER‐resident proteins ‐ SWEET talking the COPs. J Cell Sci 133, 250100. doi: 10.1242/jcs.250100 PMC7561476 33037041

[26] Buser DP , Bader G and Spiess M (2022) Retrograde transport of CDMPR depends on several machineries as analyzed by sulfatable nanobodies. Life Sci Alliance 5, e202101269. doi: 10.26508/lsa.202101269 PMC8961009 35314489

[27] Baeuerle PA and Huttner WB (1987) Tyrosine sulfation is a trans‐Golgi‐specific protein modification. J Cell Biol 105, 2655–2664. 10.1083/jcb.105.6.2655 PMC2114704 3121635

[28] Spiess M , Schwartz AL and Lodish HF (1985) Sequence of human asialoglycoprotein receptor cDNA. An internal signal sequence for membrane insertion. J Biol Chem 260, 1979–1982. 2982798

[29] Ecard J , Lian YL , Divoux S , Gouveia Z , Vigne E , Perez F and Boncompain G (2024) Lysosomal membrane proteins LAMP1 and LIMP2 are segregated in the Golgi apparatus independently of their clathrin adaptor binding motif. Mol Biol Cell 35, ar42. doi: 10.1091/mbc.E23-06-0251 PMC10916873 38231876

[30] Seaman MN (2004) Cargo‐selective endosomal sorting for retrieval to the Golgi requires retromer. J Cell Biol 165, 111–122. doi: 10.1083/jcb.200312034 PMC2172078 15078902

[31] Mayor S , Sabharanjak S and Maxfield FR (1998) Cholesterol‐dependent retention of GPI‐anchored proteins in endosomes. EMBO J 17, 4626–4638. doi: 10.1093/emboj/17.16.4626 PMC1170792 9707422

[32] Mayor S and Maxfield FR (1995) Insolubility and redistribution of GPI‐anchored proteins at the cell surface after detergent treatment. Mol Biol Cell 6, 929–944. doi: 10.1091/mbc.6.7.929 PMC301249 7579703

[33] Mayor S , Rothberg KG and Maxfield FR (1994) Sequestration of GPI‐anchored proteins in caveolae triggered by cross‐linking. Science 264, 1948–1951. doi: 10.1126/science.7516582 7516582

[34] Fasimoye R , Dong W , Nirujogi RS , Rawat ES , Iguchi M , Nyame K , Phung TK , Bagnoli E , Prescott AR , Alessi DR *et al*. (2023) Golgi‐IP, a tool for multimodal analysis of Golgi molecular content. Proc Natl Acad Sci USA 120, e2219953120. doi: 10.1073/pnas.2219953120 PMC10193996 37155866

[35] Abu‐Remaileh M , Wyant GA , Kim C , Laqtom NN , Abbasi M , Chan SH , Freinkman E and Sabatini DM (2017) Lysosomal metabolomics reveals V‐ATPase‐ and mTOR‐dependent regulation of amino acid efflux from lysosomes. Science 358, 807–813. doi: 10.1126/science.aan6298 PMC5704967 29074583

[36] Hundley FV , Gonzalez‐Lozano MA , Gottschalk LM , Cook ANK , Zhang J , Paulo JA and Harper JW (2024) Endo‐IP and lyso‐IP toolkit for endolysosomal profiling of human‐induced neurons. Proc Natl Acad Sci USA 121, e2419079121. doi: 10.1073/pnas.2419079121 PMC11670117 39636867

[37] Itzhak DN , Tyanova S , Cox J and Borner GH (2016) Global, quantitative and dynamic mapping of protein subcellular localization. elife 5, 16950. doi: 10.7554/eLife.16950 PMC4959882 27278775

[38] Hirst J , Hesketh GG , Gingras AC and Robinson MS (2021) Rag GTPases and phosphatidylinositol 3‐phosphate mediate recruitment of the AP‐5/SPG11/SPG15 complex. J Cell Biol 220, e202002075. doi: 10.1083/jcb.202002075 PMC7814351 33464297

[39] Franziscus CA , Ferrand A , Biehlmaier O , Schmidt A and Spang A (2026) SPEx: compartment‐resolved proteomics via expansion microscopy–guided microdissection. elife 15, RP111393. doi: 10.7554/eLife.111393.1

[40] Voss M (2024) Proteolytic cleavage of Golgi glycosyltransferases by SPPL3 and other proteases and its implications for cellular glycosylation. Biochim Biophys Acta Gen Subj 1868, 130668. doi: 10.1016/j.bbagen.2024.130668 38992482

[41] Buser DP , Schleicher KD , Junne T and Biehlmaier O (2025) Live‐cell imaging of endocytic transport using functionalized nanobodies in cultured cells. *J Vis Exp*, 10.3791/69284 41182966

[42] Podinovskaia M , Prescianotto‐Baschong C , Buser DP and Spang A (2021) A novel live‐cell imaging assay reveals regulation of endosome maturation. elife 10, 70982. doi: 10.7554/eLife.70982 PMC8635980 34846303

