# Supplementary material for: Golgi enzymes are retrieved from the plasma membrane to the trans‐Golgi network
Source: FEBS Lett. 2026 Aug 15;600(20):3051–72. doi: 10.1002/1873-3468.70438 (PMC13703833; doi:10.1002/1873-3468.70438)
Supplement: Supplementary file 1 [file FEB2-600-3051-s001.pdf]

Supporting Information

Golgi enzymes are retrieved from the plasma membrane to the trans-Golgi network

Dominik P. Buser and Tina Junne

Biozentrum, University of Basel, Spitalstrasse 41, CH-4056 Basel, Switzerland

Corresponding author: dominik-pascal.buser@unibas.ch
Author ORCID Buser DP: https://orcid.org/0000-0001-6019-2188

Running title: Golgi enzyme retrieval to the TGN

Content:

Figure S1. Expression of Golgi enzymes and localization of EGFP-tagged CDMPR, CNX, and ERGIC53.
Figure S2. Expression and localization of additional EGFP reporter proteins.
Figure S3. VHH-tev-mCherry for quantitative colocalization of EGFP reporter proteins.
Figure S4. Fluorescent nanobody uptake by reporter and cytosolic tail-truncated Golgi enzymes.
Figure S5. Expression, localization, and retrograde transport of full-length recombinant GalT, SiaT, and ManII.
Figure S6. Expression, localization, and retrograde transport of recombinant MGAT2, Man1B1, MGAT1, FucT, and B4GalT7.
Figure S7. Golgi-IP and Lyso-IP method reveal compartment-delivered nanobody by TPST1, TPST2, and MGAT2 from the cell surface.
Figure S8. Expression, localization, and retrograde transport of recombinant GOLM1 and GALNT2.

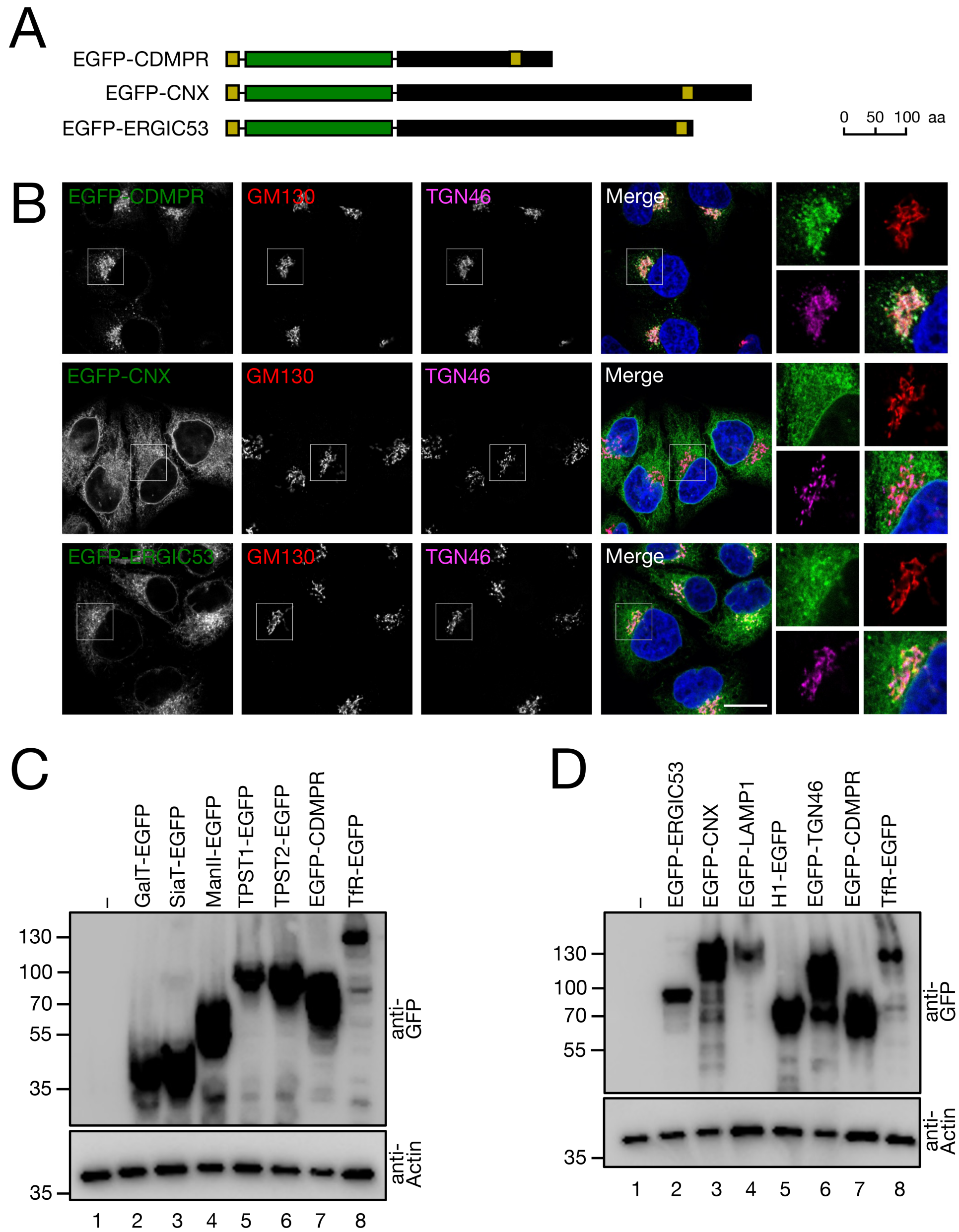

Figure S1. Expression of Golgi enzymes and localization of EGFP-tagged CDMPR, CNX, and ERGIC53.

(**A**) Schematic representation of EGFP (green) fusion proteins. Sequences derived from the reporter protein are shown in black with signal sequence and internal transmembrane segments in yellow. EGFP was fused N-terminally to full-length CDMPR, CNX, and ERGIC53. Scale bar in amino acids (aa).

(**B**) HeLa cells stably expressing EGFP-CDMPR, -CNX, or -ERGIC53 were fixed, stained for GM130, TGN46, and nuclei (DAPI, blue), and imaged by fluorescence microscopy. Insets display enlarged regions of the selected area. For figure preparation, brightness was adjusted for visualization only. When applicable, all quantitative analyses were performed using the original, unprocessed image data. Bar: 20 μm.

(**C, D**) HeLa cells stably expressing EGFP-tagged Golgi enzymes and reporter proteins were analyzed by SDS-gel electrophoresis and immunoblotting using anti-GFP and -actin antibodies. The positions of size markers with molecular mass in kiloDaltons are indicated.

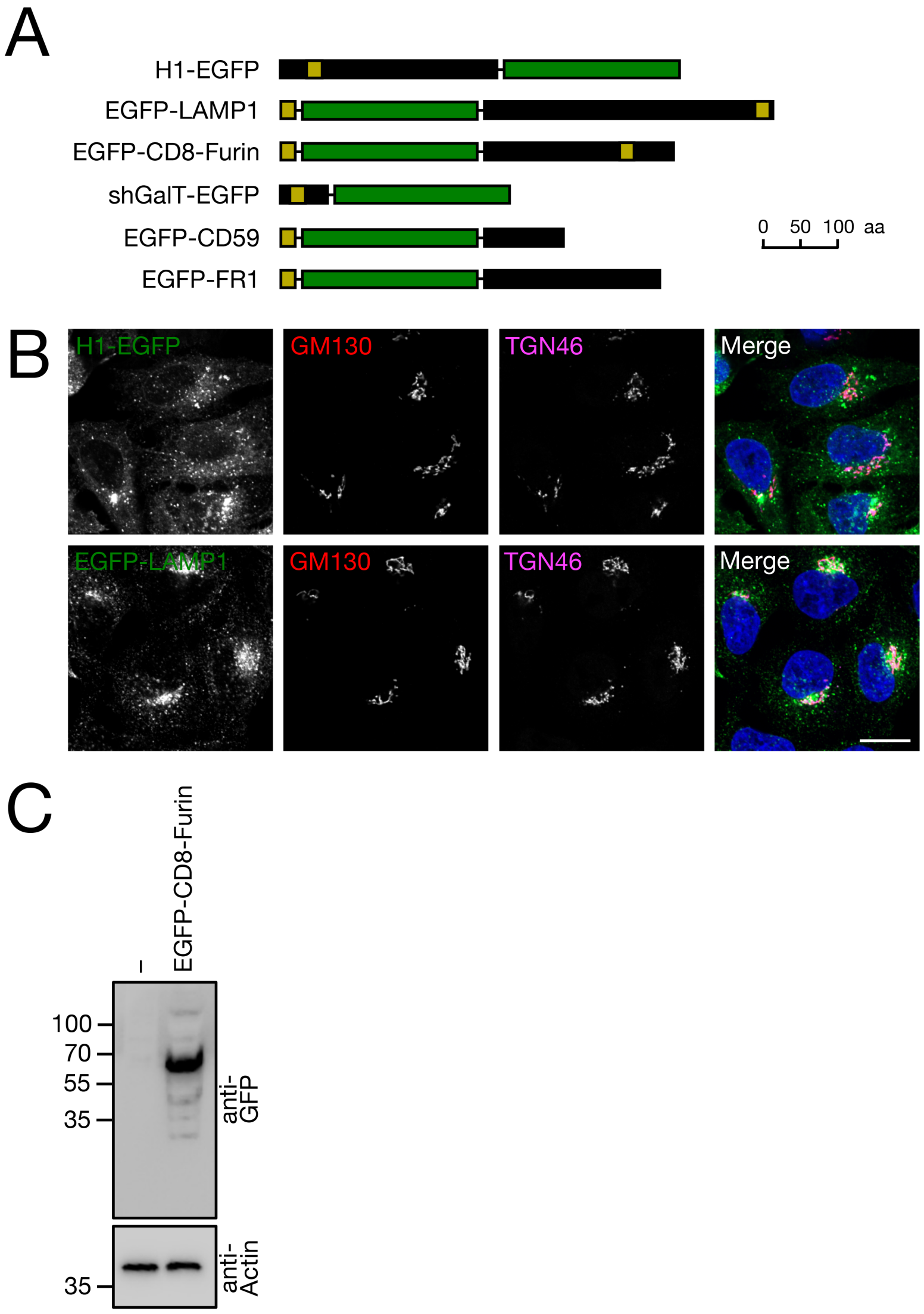

Figure S2. Expression and localization of additional EGFP reporter proteins.

(**A**) Schematic representation of EGFP (green) fusion proteins. Sequences derived from the reporter protein are shown in black with signal sequence and internal transmembrane segments in yellow. EGFP was fused C-terminally to full-length H1 and short GalT (shGalT), and N-terminally to full-length LAMP1, CD8-Furin, CD59, and FR1. Scale bar in amino acids (aa).

(**B**) HeLa cells stably expressing H1-EGFP or EGFP-LAMP1 were fixed, stained for GM130, TGN46, and nuclei (DAPI, blue), and imaged by fluorescence microscopy. For figure preparation, brightness was adjusted for visualization only. When applicable, all quantitative analyses were performed using the original, unprocessed image data. Bar: 20 µm.

(**C**) HeLa cells stably expressing EGFP-CD8-Furin were analyzed by SDS-gel electrophoresis and immunoblotting using anti-GFP and -actin antibodies. The positions of size markers with molecular mass in kiloDaltons are indicated.

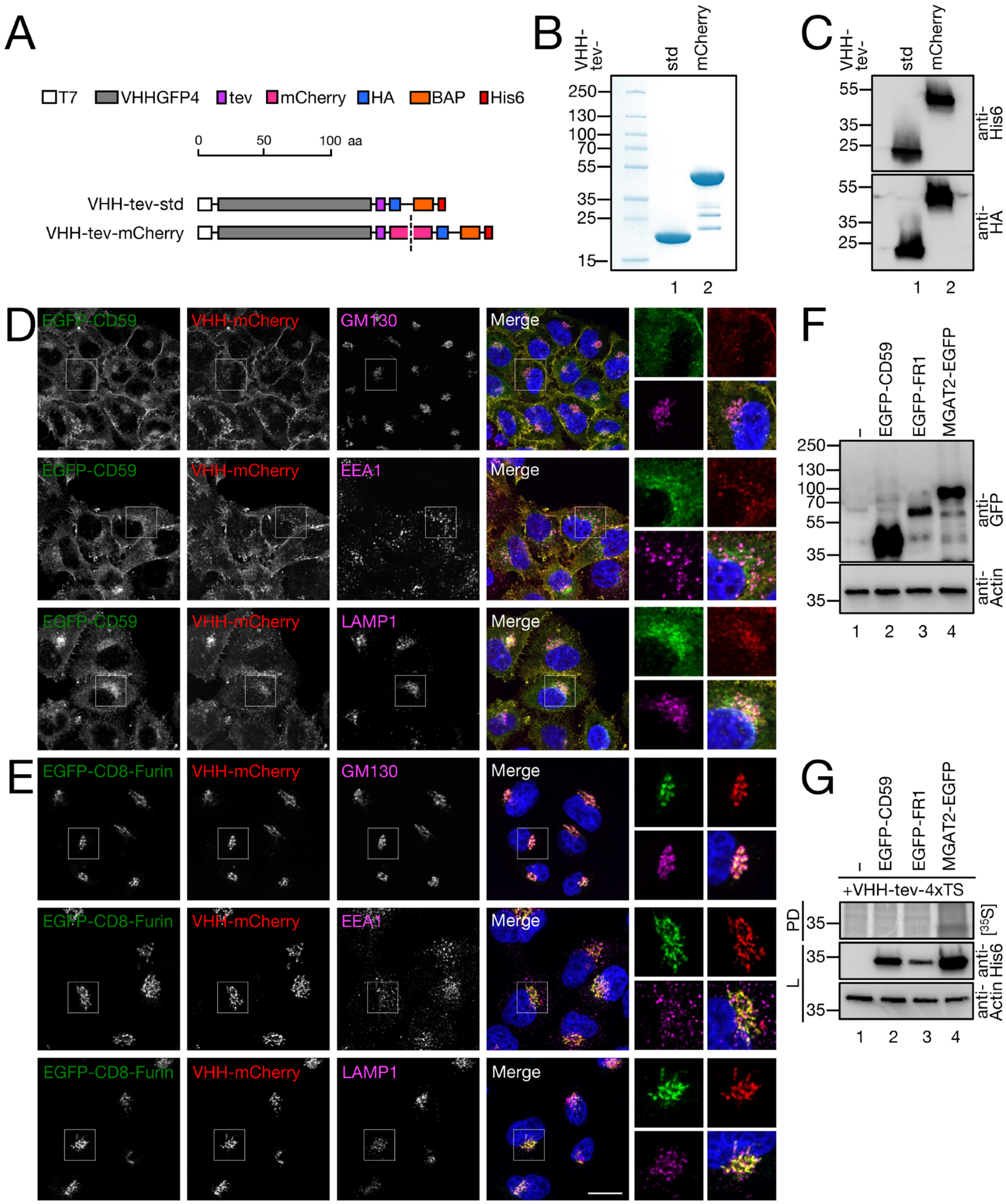

Figure S3. VHH-tev-mCherry for quantitative colocalization of EGFP reporter proteins.

(**A**) Schematic representation of the derivatized fluorescent nanobody VHH-tev-mCherry. The modified standard nanobody (VHH-tev-std) consists of the GFP-specific VHH domain, T7 and HA epitope tags, a TEV cleavage site, a BAP, and a hexahistidine (His6) purification tag. VHH-tev-mCherry contains mCherry where the sulfate nanobody contains four copies of tyrosine sulfation consensus sequence. The dashed line within mCherry denotes that the complete

protein sequence (236 aa) is not fully represented. Scale bar is in amino acids (aa).

(B) Bacterially expressed and purified nanobodies (30 μg) were analyzed by SDS-gel electrophoresis and Coomassie stained. Marker proteins with molecular mass in kiloDaltons are shown on the left.

(C) Immunoblot analysis of nanobodies (30 ng) with antibodies against the HA or His6 epitopes. Marker proteins with molecular mass in kiloDaltons are shown on the left.

(**D, E**) HeLa cells stably expressing the indicated EGFP-tagged membrane proteins were allowed to endocytose VHH-tev-mCherry (at 5 μg/ml) for 2 h (CD8-Furin) or 5 h (CD59), fixed, stained for GM130, EEA1, or LAMP1, and nuclei (DAPI, blue), and imaged by fluorescence microscopy. Insets display enlarged regions of the selected area. For figure preparation, brightness was adjusted for visualization only. When applicable, all quantitative analyses were performed using the original, unprocessed image data. Bar: 20 μm.

(**F**) HeLa cells stably expressing EGFP-CD59, EGFP-FR1, and MGAT2-EGFP were analyzed by SDS-gel electrophoresis and immunoblotting using anti-GFP and -actin antibodies. The positions of size markers with molecular mass in kiloDaltons are indicated.

(**G**) HeLa cells stably expressing the indicated EGFP-tagged membrane proteins were labeled with [^{35}S]sulfate for 5 h with 5 μg/ml VHH-tev-4xTS. HeLa expressing no reporter protein was processed as control. The nanobodies were isolated using Nickel resin-based pulldowns (PD) and subjected to autoradiography ([^{35}S]). A fraction of the postnuclear supernatant of lysed cells after labeling was subjected to immunoblot analysis. Aliquots of collected postnuclear cell lysates (L) were immunoblotted for His6 (total cell-associated nanobody) and actin (loading control). There was sulfated nanobody observed for MGAT2, only.

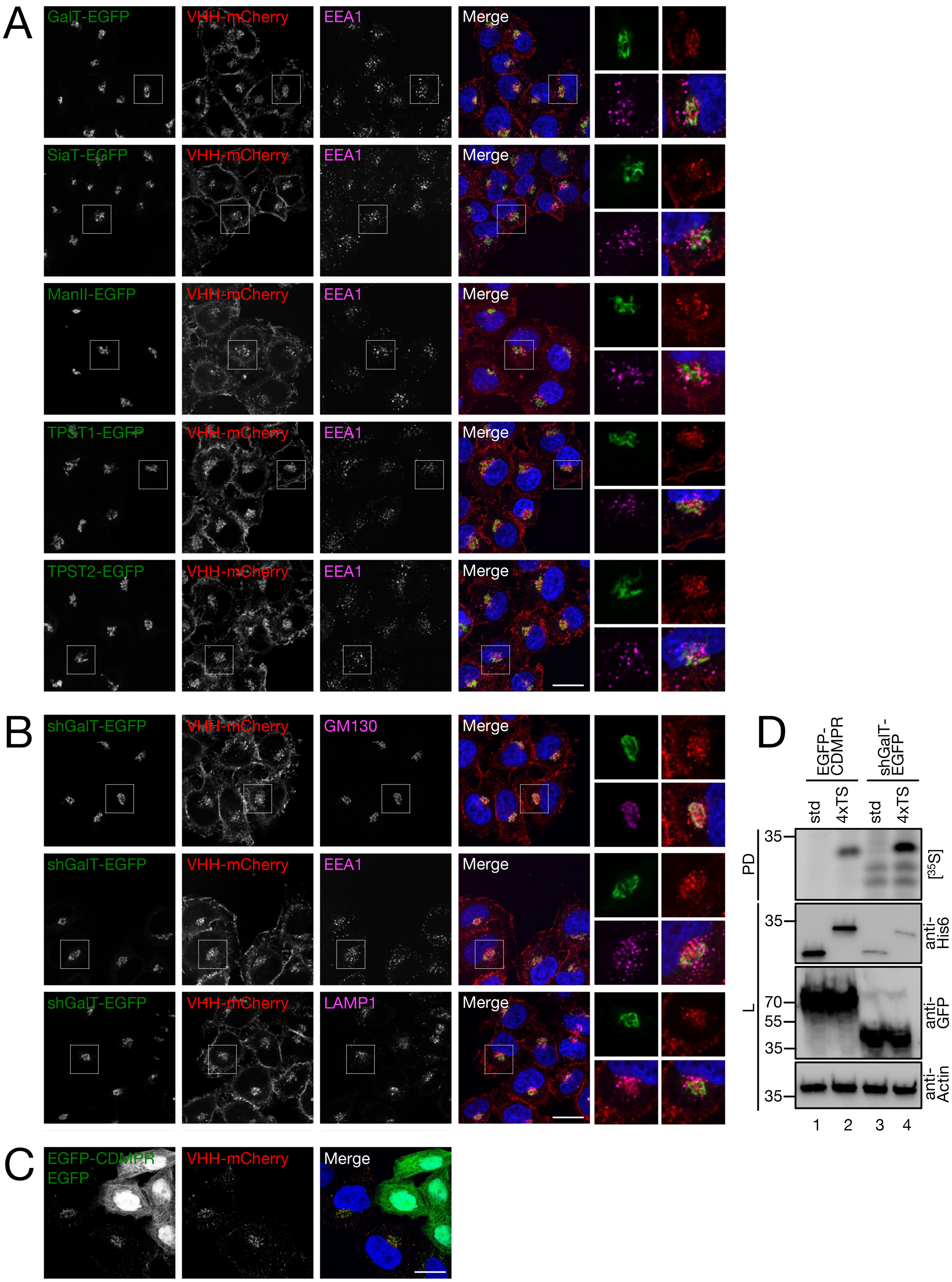
A
GalT-EGFP
VHH-mCherry
EEA1
Merge
SiaT-EGFP
ManII-EGFP
TPST1-EGFP
TPST2-EGFP
B
shGalT-EGFP
GM130
LAMP1
C
EGFP-CDMPR
EGFP
D
EGFP-CDMPR
shGalT-EGFP
std
4xTS
PD
L
35
70
55
[35S]
anti-His6
anti-GFP
anti-Actin
1
2
3
4

Figure S4. Fluorescent nanobody uptake by reporter and cytosolic tail-truncated Golgi enzymes.

(**A**) HeLa cells stably expressing the indicated EGFP-tagged Golgi enzymes were allowed to endocytose VHH-tev-mCherry (at 5 μg/ml) for 5 h, fixed, stained for EEA1 and nuclei (DAPI, blue), and imaged by fluorescence microscopy. Insets display enlarged regions of the selected area. For figure preparation, brightness was adjusted for visualization only. When applicable, all quantitative analyses were performed using the original, unprocessed image data. Bar: 20 μm.

(**B**) HeLa cells stably expressing shGalT-EGFP were allowed to endocytose VHH-tev-mCherry (at 5 μg/ml) for 5 h, fixed, stained for GM130, EEA1, or LAMP1, and nuclei (DAPI, blue), and imaged by fluorescence microscopy. Insets display enlarged regions of the selected area. For figure preparation, brightness was adjusted for visualization only. When applicable, all quantitative analyses were performed using the original, unprocessed image data. Bar: 20 μm.

(**C**) Proof-of-concept that nanobody can be only endocytosed when reporter proteins transiently expose EGFP on their extracellular domain. HeLa cells stably expressing the EGFP or EGFP-CDMR were mixed and seeded, followed by fixation, staining for nuclei (DAPI, blue), and imaging by fluorescence microscopy. For figure preparation, brightness was adjusted for visualization only. When applicable, all quantitative analyses were performed using the original, unprocessed image data. Bar: 20 μm.

(**D**) HeLa cells stably expressing EGFP-CDMPR or shGalT-EGFP were labeled with [^{35}S]sulfate for 1 h with 2 μg/ml VHH-tev-std or VHH-tev-4xTS. The nanobodies were isolated using Nickel resin-based pulldowns (PD) and subjected to autoradiography ([^{35}S]). A fraction of the postnuclear supernatant of lysed cells after labeling was subjected to immunoblot analysis. Aliquots of collected postnuclear cell lysates (L) were immunoblotted for His6 (total cell-associated nanobody), GFP (reporter expression), and actin (loading control).

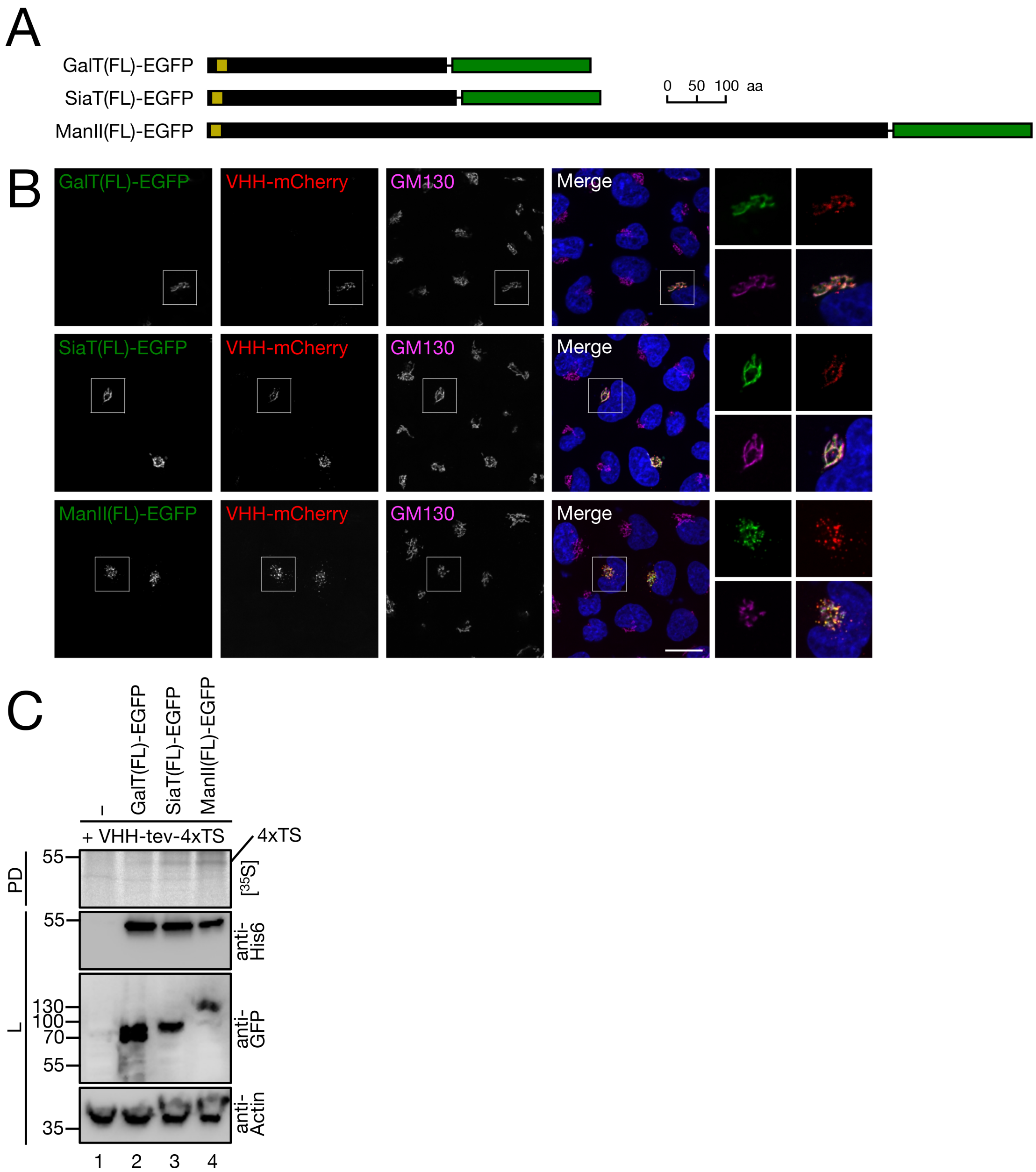

Figure S5. Expression, localization, and retrograde transport of full-length recombinant GalT, SiaT, and ManII.

(**A**) Schematic representation of EGFP (green) fusion proteins. Sequences derived from the reporter protein are shown in black with signal sequence and internal transmembrane segments in yellow. EGFP was fused C-terminally to full-length GalT, SiaT, and ManII. Scale bar in amino acids (aa).

(**B**) HeLa cells transiently expressing full-length GalT, SiaT, and ManII were allowed to endocytose VHH-tev-mCherry (at 5 µg/ml) for 5 h, fixed, stained for GM130 and nuclei (DAPI, blue), and imaged by fluorescence microscopy. Insets display enlarged regions of the selected area. For figure preparation, brightness was adjusted for visualization only. When applicable, all quantitative analyses were performed using the original, unprocessed image data. Bar: 20 µm.

(**C**) HeLa cells transiently expressing full-length GalT, SiaT, and ManII were labeled with [^{35}S]sulfate for 5 h with 2 µg/ml VHH-tev-4xTS. HeLa expressing no reporter protein was processed as control. The nanobodies were isolated using

Nickel resin-based pulldowns (PD) and subjected to autoradiography ([^{35}S]). A fraction of the postnuclear supernatant of lysed cells after labeling was subjected to immunoblot analysis. Aliquots of collected postnuclear cell lysates (L) were immunoblotted for His6 (total cell-associated nanobody), GFP (reporter expression), and actin (loading control).

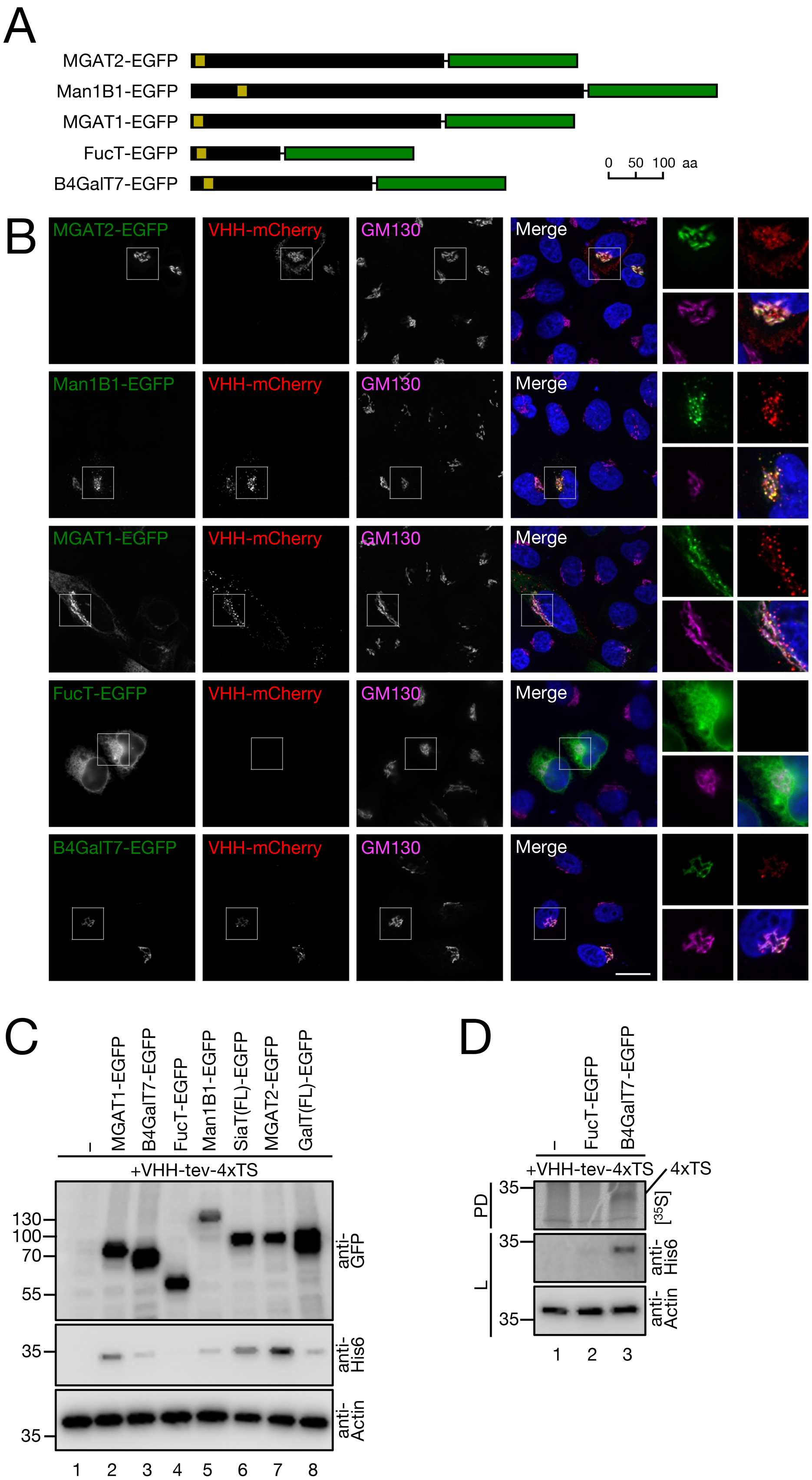
A
MGAT2-EGFP
Man1B1-EGFP
MGAT1-EGFP
FucT-EGFP
B4GalT7-EGFP
0 50 100 aa
B
MGAT2-EGFP
VHH-mCherry
GM130
Merge
Man1B1-EGFP
MGAT1-EGFP
FucT-EGFP
B4GalT7-EGFP
C
MGAT1-EGFP
B4GalT7-EGFP
FucT-EGFP
Man1B1-EGFP
SiaT(FL)-EGFP
MGAT2-EGFP
GalT(FL)-EGFP
+VHH-tev-4xTS
130
100
70
55
35
anti-GFP
anti-His6
anti-Actin
1 2 3 4 5 6 7 8
D
FucT-EGFP
B4GalT7-EGFP
+VHH-tev-4xTS
4xTS
PD
L
[35S]
1 2 3

Figure S6. Expression, localization, and retrograde transport of recombinant MGAT2, Man1B1, MGAT1, FucT, and B4GalT7.

(**A**) Schematic representation of EGFP (green) fusion proteins. Sequences derived from the reporter protein are shown in black with signal sequence and internal transmembrane segments in yellow. EGFP was fused C-terminally present at the reporter. Scale bar in amino acids (aa).

(**B**) HeLa cells transiently expressing EGFP-tagged MGAT2, Man1B1, MGAT1, FucT, B4GalT7 were allowed to endocytose VHH-tev-mCherry (at 5 µg/ml) for 5 h, fixed, stained for GM130 and nuclei (DAPI, blue), and imaged by fluorescence microscopy. MGAT2 was used as control and was already assayed in Fig.4. Insets display enlarged regions of the selected area. For figure preparation, brightness was adjusted for visualization only. When applicable, all quantitative analyses were performed using the original, unprocessed image data. Bar: 20 µm.

(**C**) HeLa cells transiently expressing EGFP-tagged Golgi enzymes were allowed to endocytose VHH-tev-std (at 5 µg/ml) for 1 h, fixed were analyzed by SDS-gel electrophoresis and immunoblotting using anti-GFP, -actin, and -His6 antibodies. The positions of size markers with molecular mass in kiloDaltons are indicated.

(**D**) HeLa cells transiently expressing FucT-EGFP or B4GalT7-EGFP were labeled with [^{35}S]sulfate for 5 h with 2 µg/ml VHH-tev-4xTS. HeLa expressing no reporter protein was processed as control. The nanobodies were isolated using Nickel resin-based pulldowns (PD) and subjected to autoradiography ([^{35}S]). A fraction of the postnuclear supernatant of lysed cells after labeling was subjected to immunoblot analysis. Aliquots of collected postnuclear cell lysates (L) were immunoblotted for His6 (total cell-associated nanobody) and actin (loading control).

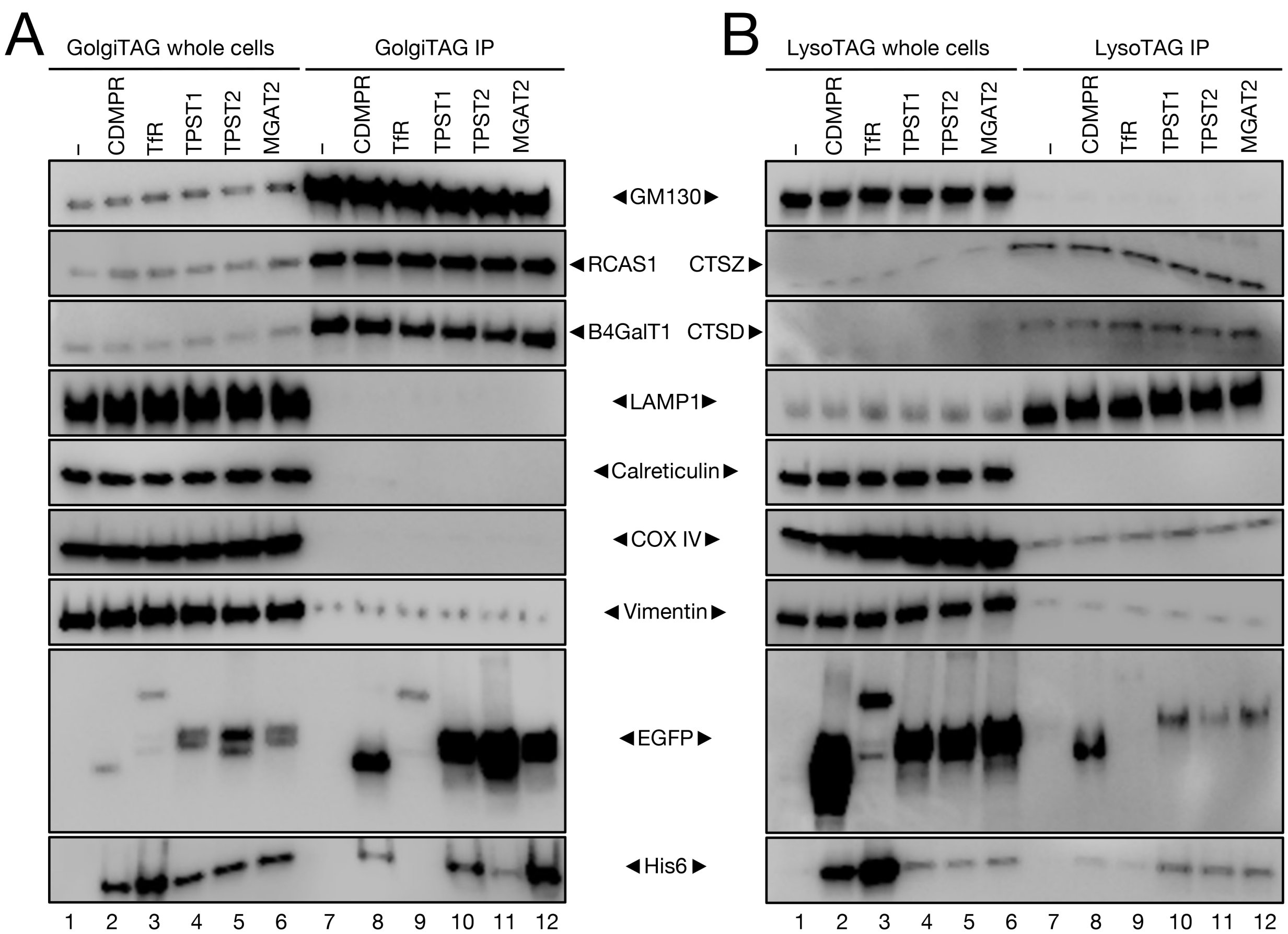

Figure S7. Golgi-IP and Lyso-IP method reveal compartment-delivered nanobody by TPST1, TPST2, and MGAT2 from the cell surface.

(**A**) Immunoblotting for protein markers of various subcellular compartments in whole cell lysates, and purified Golgi using Golgi-IP. The Golgi-IP isolates pure Golgi membranes. GolgiTAG cells were transfected with plasmids encoding EGFP-fusions of CDMPR, TfR, TPST1, TPST2, and MGAT2, followed by uptake with nanobody (VHH-tev-4xTS). Untransfected GolgiTAG cells incubated with nanobody served as control. Whole cell lysates as well as the resuspended immunoprecipitates (IPs) were subjected to immunoblotting with the indicated compartment-specific antibodies, among them are three Golgi-specific markers: GM130, RCAS1, and B4GalT1. Anti-His6 detects VHH-tev-4xTS in whole cell lysates or IP.

(**B**) Immunoblotting for protein markers of various subcellular compartments in whole cell lysates, and purified lysosomes using Lyso-IP. The Lyso-IP isolates pure lysosomes. LysoTAG cells were transfected with plasmids encoding EGFP fusions of CDMPR, TfR, TPST1, TPST2, and MGAT2, followed by uptake with nanobody (VHH-tev-4xTS). Untransfected LysoTAG cells incubated with nanobody served as control. Whole cell lysates as well as the resuspended immunoprecipitates (IPs) were subjected to immunoblotting with the indicated compartment-specific antibodies, among them are three lysosome-specific markers: LAMP1, CTSD, and CTSZ. Anti-His6 detects VHH-tev-4xTS in whole cell lysates or IP.

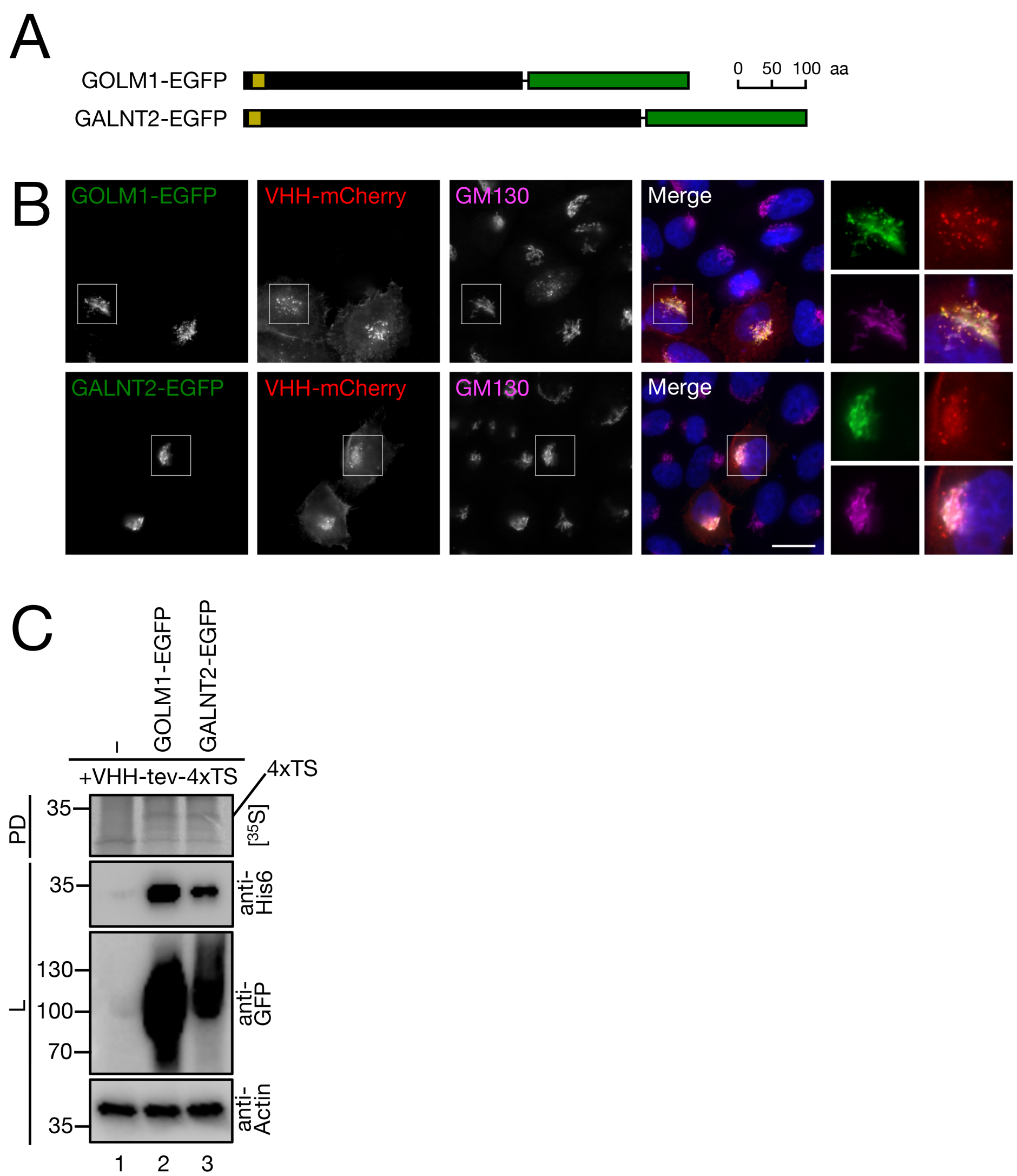

Figure S8. Expression, localization, and retrograde transport of recombinant GOLM1 and GALNT2.

(**A**) Schematic representation of EGFP (green) fusion proteins. Sequences derived from the reporter protein are shown in black with signal sequence and internal transmembrane segments in yellow. EGFP was fused C-terminally GOLM1 and GALNT2. Scale bar in amino acids (aa).

(**B**) HeLa cells transiently expressing EGFP-tagged GOLM1 or GALNT2 were allowed to endocytose VHH-tev-mCherry (at 5 µg/ml) for 5 h, fixed, stained for GM130 and nuclei (DAPI, blue), and imaged by fluorescence microscopy. Insets display enlarged regions of the selected area. For figure preparation, brightness was adjusted for visualization only. When applicable, all quantitative analyses were performed using the original, unprocessed image data. Bar: 20 µm.

(**C**) HeLa cells transiently expressing GOLM1-EGFP or GALNT2-EGFP were labeled with [^{35}S]sulfate for 5 h with 2 µg/ml VHH-tev-4xTS. HeLa expressing no reporter protein was processed as control. The nanobodies were isolated using Nickel resin-based pulldowns (PD) and subjected to autoradiography ([^{35}S]). A fraction of the postnuclear supernatant of lysed cells after labeling was subjected to immunoblot analysis. Aliquots of collected postnuclear cell lysates (L) were immunoblotted for His6 (total cell-associated nanobody) and actin (loading control).
